# Fatty acid and nutrient profiles, diosgenin and trigonelline contents, mineral composition, and antioxidant activity of the seed of some Iranian *Trigonella* L. species

**DOI:** 10.1186/s12870-024-05341-9

**Published:** 2024-07-15

**Authors:** Ziba Bakhtiar, Mohammadreza Hassandokht, Mohammad Reza Naghavi, Hassan Rezadoost, Mohammad Hossein Mirjalili

**Affiliations:** 1 Department of Horticultural Sciences, College of Agriculture and Natural Resources, University of Tehran, Karaj, Iran; 2 Division of Biotechnology, Department of Agronomy and Plant Breeding, College of Agriculture and Natural Resources, University of Tehran, Karaj, Iran; 3 Department of Phytochemistry, Medicinal Plants and Drugs Research Institute of Shahid Beheshti University, Tehran, 1983969411 Iran; 4 Department of Agriculture, Medicinal Plants and Drugs Research Institute, Shahid Beheshti University, Tehran, 1983969411 Iran

**Keywords:** Fatty acid, Mineral, Natural products, Proximate composition, Phenol

## Abstract

**Background:**

Fenugreeks (*Trigonella* L. spp.), belonging to the legume family (Fabaceae), are well-known multipurpose crops that their materials are currently received much attention in the pharmaceutical and food industries for the production of healthy and functional foods all over the world. Iran is one of the main diversity origins of this valuable plant. Therefore, the aim of the present study was to explore vitamins, minerals, and fatty acids profile, proximate composition, content of diosgenin, trigonelline, phenolic acids, total carotenoids, saponins, phenols, flavonoids, and tannins, mucilage and bitterness value, and antioxidant activity of the seed of thirty populations belonging to the ten different Iranian *Trigonella* species.

**Results:**

We accordingly identified notable differences in the nutrient and bioactive compounds of each population. The highest content (mg/100 g DW) of ascorbic acid (18.67 ± 0.85‒22.48 ± 0.60) and α–tocopherol (31.61 ± 0.15‒38.78 ± 0.67) were found in the populations of *T. filipes* and *T. coerulescens*, respectively. Maximum content of catechin was found in the populations of *T. teheranica* (52.67 ± 0.05‒63.50 ± 0.72 mg/l). Linoleic acid (> 39.11% ± 0.61%) and linolenic acid (> 48.78 ± 0.39%) were the main polyunsaturated fatty acids, with the majority in the populations of *T. stellata* (54.81 ± 1.39‒63.46 ± 1.21%). The populations of *T. stellata* were also rich in trigonelline (4.95 ± 0.03‒7.66 ± 0.16 mg/g DW) and diosgenin (9.06 ± 0.06‒11.03 ± 0.17 mg/g DW).

**Conclusions:**

The obtained data provides baseline information to expand the inventory of wild and cultivated Iranian *Trigonella* species for further exploitation of rich chemotypes in the new foods and specific applications.

**Supplementary Information:**

The online version contains supplementary material available at 10.1186/s12870-024-05341-9.

## Background

Throughout history, human societies have developed a wide variety of dietary patterns from available plants and animals. Today, the industrialized countries of the world also recommend diets that are mainly based on plant sources. Plant-based foods are important sources of energy, protein, vitamins, minerals, and fiber in the human diet [1, 2].

In addition to increase the production of plant-based foods, quality is also a focus in improving food products. So, not only the volume of food but also its ingredients affect human health [3]. Therefore, increasing the production of crops and introducing new plant sources with high nutritional value is the most important issue in agriculture that pays attention by all countries [4].

The recent development of functional foods and pharmaceutical products based on medicinal and food plants rich in natural antioxidants such as phenolic acids, flavonoids, anthocyanins, and tannins [5], has reduced the use of synthetic drugs. Many efforts have been made to find natural antioxidants from plant sources so far. This process has been done for many agricultural crops including fruits, edible seeds, vegetables, and cereals since, a long time ago by selection and breeding of species, cultivars, wild populations, accessions, and other vegetation based on the nutritional, phytochemical, and mineral characteristics [6].

The nutritional value of many agricultural crops including wheat, corn, potato, beet, and canola [3], has been increased by breeding programs. Improving nutritional and phytochemical value and obtaining quality products have also been of great interest [7–10]. Protein, carbohydrates, vitamins, unsaturated fats, antioxidant compounds, and crude fiber are among the most important nutritional factors considered when selecting and introducing plant foods.

More than 820 million people suffer from insufficient food and protein deficiency. Compared to other crops, including cereals, legumes offer a high protein level in their biomass and underground organs [11]. The detrimental environmental impact of meat production, ethical concerns regarding animal rights, and health considerations have led to a growing interest in the production of legume-based plant foods as a protein source for human consumption [12]. Additionally, the ability of legumes to fix atmospheric nitrogen makes their cultivation a sustainable option, reducing the reliance on chemical fertilizers, production costs, and greenhouse gas emissions [13].

According to the Legume Phylogeny Working Group [14], 400,000 plant species are approximately growing on the earth, of which 5% are members of the Fabaceae family. These plants are known as legumes by producing protein-rich seeds. This family includes 770 genera and approximately 20,000 species distributed worldwide [14]. Beans (*Vicia faba* L.), peas (*Pisum sativum* L.), mung bean (*Vigna radiata* L.), soybean (*Glycine max* (L.) Merr.), green bean (*Phaseolus vulgaris* L.), peanut (*Arachis hypogaea* L.), lupine (*Lupinus* spp.), alfalfa (*Medicago sativa* L.), clover (*Trifolium* spp.) and fenugreeks (*Trigonella* L. spp.) are the major legume crops that are widely cultivated across the world [15].

Among 70 *Trigonella* species that are distributed throughout the world, *T. arabica* Delile, *T. caerulea* (L.) Ser., *T. corniculata* Sibth. & Sm., *T. stellata* Forssk., and *T. foenum-graecum* L. are the most important species [16]. Twenty *Trigonella* species are reported in the flora of Iran [17]. *Trigonella foenum-graecum* (Fenugreek) is a well-known species that is a native plant found in the parts of Iran to the North of India and grows in most parts of the world including Ethiopia, Canada, Oman, and Turkey [18, 19]. The plant is one of the most important medicinal plants and vegetables that are widely cultivated in the world for food purposes.

Fenugreek seeds with a golden yellow color, hard and tetrahedral structure are common and valuable parts of the plant that are traditionally used in the treatment of cough and cold and antipyretic [11]. Cornbread mixed with a small amount of fenugreek (3%) is known as the main food in Egypt [20]. In Italian recipes, fenugreek seeds are also used to flavor pizza and special pasta [21]. The biological and medicinal effects of fenugreek, including the impacts of reducing fat, sugar and blood pressure, protecting the liver and stomach, as well as antioxidant activity, are mostly attributed to the variety of its bioactive chemical compounds. The plant is also a raw material for making various hormonal and therapeutic drugs [22, 23].

Various natural compounds including flavonoids, alkaloids (*i.e.* trigonelline), steroidal saponins (*i.e.* diosgenin), tannins, and phenolics in the plant seeds have also been reported [19, 23–26]. Fenugreek seeds contain high amounts of important nutrients, vitamins, mucilage, protein and fat [27–29].

Research on the conservation of genetic resources, plant production, and utilization, including commercial cultivation, as well as the analysis of food and natural compounds for various industrial applications, is crucial. In this context, the active ingredients and nutritional components of *T. foenum-graecum* from Iran, India, Tunisia, Germany, Algeria, and Malaysia have been extensively investigated [26, 28, 30–36]. Based on previous reports, wild species of *Trigonella* from Turkey have been found to possess high nutritional value and contain essential minerals and natural compounds [37, 38]. However, information on other *Trigonella* species is currently limited. In addition, quantitative and qualitative assessment of nutritional and phytochemical traits of Iranian *Trigonella* species has not been investigated so far.

Given the nutritional and medicinal significance of fenugreek, the purpose of the present study was to evaluate the fatty acid and nutrient profiles, diosgenin and trigonelline contents, mineral composition, and antioxidant activity of ten Iranian *Trigonella* species under uniform cultivation conditions. We hypothesized that wild *Trigonella* species would exhibit higher levels of these compounds. The findings serve as a foundational step in identifying superior species for potential utilization in the food and pharmaceutical industries.

## Materials and methods

### Chemicals

All chemicals used in this research were of analytical grade and were purchased from Sigma-Aldrich Co. (Buchs, Switzerland), Merck (Darmstadt, Germany), and Thermo Fisher, USA.

## Plant materials and cultivation site

**Fig. 1:**
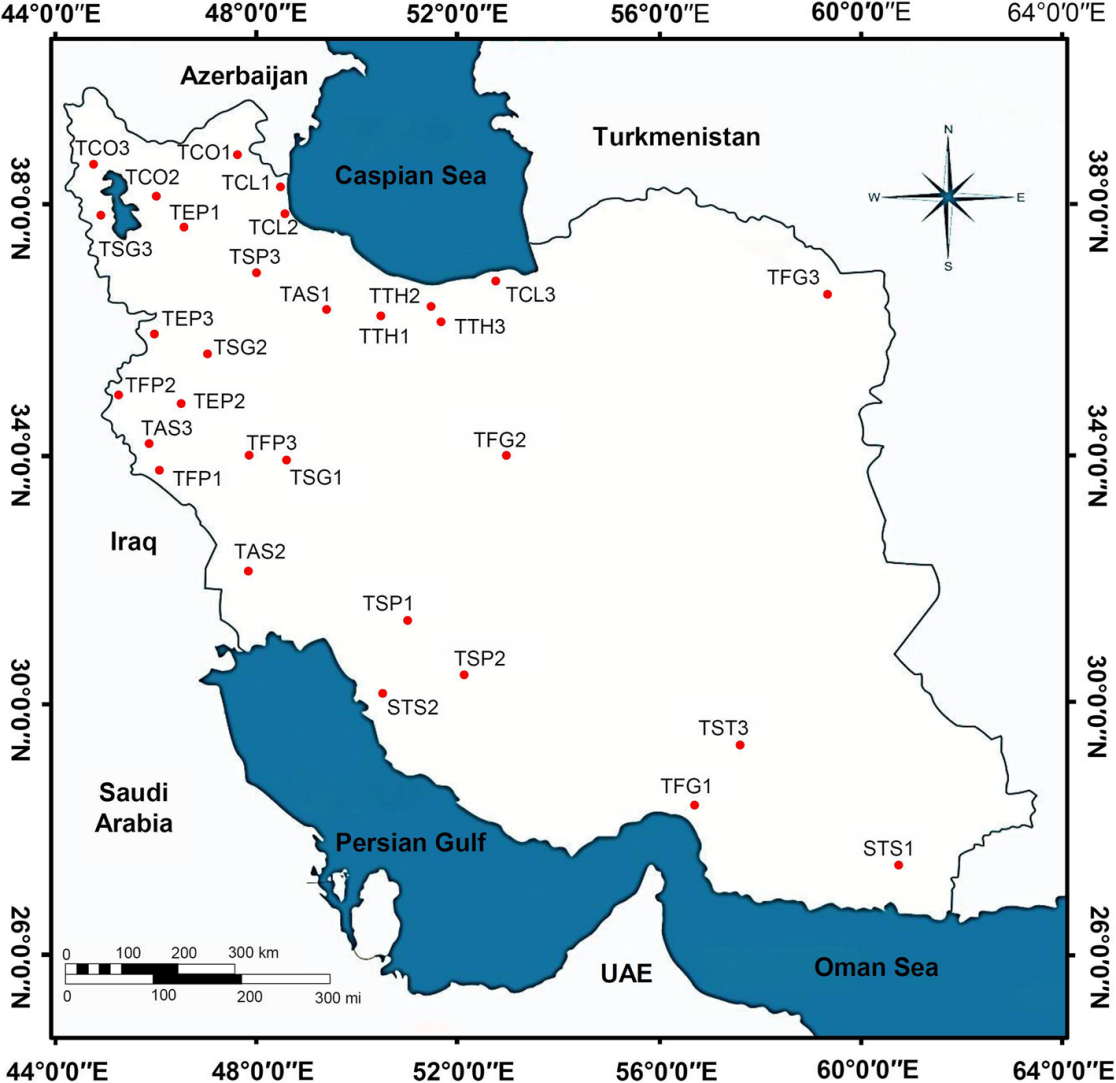
Distribution map of the thirty wild populations of ten *Trigonella* species collected across Iran. *T. astroides*1 (TAS1, Soltanabad), *T. astroides*2 (TAS2, Bavi), *T. astroides*3 (TAS3, Mehran), *T. calliceras*1 (TCL1, Astara), *T. calliceras*2 (TCL2, Bandar Anzali), *T. calliceras*3 (TCL3, Behshahr), *T. coerulescens*1 (TCO1, Meshginshahr), *T. coerulescens*2 (TCO2, Tabriz), *T. coerulescens*3 (TCO3, Khoy), *T. elliptica*1 (TEP1, Mianeh), *T. elliptica*2 (TEP2, Kermanshah), *T. elliptica*3 (TEP3, Mariwan), *T. filipes*1 (TFP1, Salehabad), *T. filipes*2 (TFP2, Qasr e Shirin), *T. filipes*3 (TFP3, Saravand), *T. foenum-graecum*1 (TFG1, Minab), *T. foenum-graecum*2 (TFG2, Ardestan), *T. foenum-graecum*3 (TFG3, Mashhad), *T. spruneriana*1 (TSP1, Pataveh), *T. spruneriana*2 (TSP2, Shiraz), *T. spruneriana*3 (TSP3, Tarom), *T. stellata*1 (TST1, Qasregand), *T. stellata*2 (TST2, Borazjan), *T. stellata*3 (TST3, Kahnuj), *T. strangulata*1 (TSG1, Khorramabad), *T. strangulata*2 (TSG2, Ghorveh), *T. strangulata*3 (TSG3, Urmia), *T. teheranica*1 (TTH1, Karaj), *T. teheranica*2 (TTH2, Chalus), and *T. teheranica*3 (TTH3, Oushan)

Seeds of thirty populations of the ten *Trigonella* species were obtained from the Iranian Biological Resource Center (IBRC), collected from different parts of Iran (Fig. 1 and Supplementary Table 1), with an altitude ranging from –28 to 2500 m. The seeds were planted in the field at the Horticultural Research Station at University of Tehran, Mohammadshahr, Karaj, Iran (N35° 46′, E50° 55′ at an altitude of 1320 m) from May to September 2021. The experiment was completely randomized block design (CRBD) as one of the standard designs with three replicates. This design was used due to one-way changes in the experimental material and providing more accurate results. The seeds of thirty wild populations of ten Iranian *Trigonella* species (each species includes three populations) were planted using 0.5 × 1 m plots with three replicates.

Due to the correct decision regarding soil amendment, fertilization management, leaching control, and energy saving, the soil physicochemical characteristics of the field were analyzed in the water and soil research laboratory at University of Tehran, Karaj, Iran, according to the standard methods. For instance, the organic carbon was determined according to the Walkley–Black method [39]. The pH of the soil extract was assessed by a potentiometric titrator (Orion Star T940, Thermo Scientific, USA). The soil texture and salinity were determined using the soil triangle and the electrical conductivity (MW301 PRO, Milwaukee, USA), respectively. Lime, phosphorus, and potassium were measured by calcium bicarbonate, colorimetrically, and sodium tetraphenyl boron methods, respectively. The soil of the field had a salinity of 0.7 ds/m, pH of 7.6, organic matter of 0.8%, lime of 5.6%, phosphorus of 21 mg/kg, and potassium of 340 mg/kg. The texture of the soil was loamy.

The weeds were controlled by hand twice, and no herbicide and fertilization were used. During the growth period, supplementary irrigation (once a week) was applied to the experimental areas to provide a water supply equivalent to an average crop growing season for the region.

The seeds were collected in the near-dry, mature state in September 2021, completely dried in the shade, and were used for the analysis. The voucher specimens have been deposited for all studied samples in Herbarium of College of Agriculture and Natural Resources (Herbarium Instituti Agronomici Keredjensis) (HIAK), University of Tehran, Karaj, Iran (Supplementary Table 1).

## Determination of vitamins

In the present study, the content of different vitamins including β-carotene, B group (B1, B2, B3, and B6), C, and E (α–tocopherol) was measured according to the following methods. β-Carotene was determined as described previously by Negi and Roy [40]. Initially, dried powdered seed sample was (2 g) mixed with acetone (10 ml), and petroleum ether (2 ml). The petroleum ether phase was passed through sodium sulfate (20 ml). Then, the extract was filtered through a column containing magnesium oxide and diatomaceous 1: 1 (w/w). Absorbance was taken at 440 nm using a spectrophotometer (Bio-Tek Instruments, Inc., USA).

All B vitamins group were calculated by Akintimehin et al. [41] and Association of Official Analytical Chemists (AOAC) [42], methods with a couple modifications. For the measurement of thiamin (vitamin B1), dried powdered seed sample (1 g) was initially added to 100 ml hydrochloric acid (0.1 N) solution and then centrifuged (Centrifuge Rotanta 460r, Hettich, Germany) at 4400 rpm for 5 min. Respectively, 5 ml ethanol (96%), potassium ferricyanide (III) solution (1% v/v), and 10 ml toluene (99.5%) were added to the solution. To determine vitamin riboflavin (vitamin B2) content, dried powdered seed sample (1 g) was mixed with 100 ml acetic acid (glacial)–water solution (50:50 v/v). The solution was then shaken and left for 30 min in a water bath at 100 °C. The solution was filtered with Whatman filter paper No. 1. Niacin (vitamin B3) was calculated with adding dried powdered seed sample (1 g) of each sample to 5 ml hydrochloric acid (5 N), 5 ml dichloromethane, and 90 ml deionized water. The mixture was boiled for 30 min at 100 ͦ C. For evaluation of pyridoxine (vitamin B3), dried powdered seed sample (1 g) was mixed with 10 ml hydrochloric acid (0.1 N) and 600 μl 4-deoxypyridoxine (100 μg/ml). The pH was adjusted to 4.5, and 1 ml takadiastase (10% w/v) was added. Then, 400 μl trichloroacetic acid (50% w/v) was added and heated for 5 min at 100 °C. The volume was made up to 20 ml with water.

The analysis for each sample was conducted in triplicate. The absorbance of thiamin, riboflavin, niacin, and pyridoxine were measured at wavelengths of 530, 461, 410, and 290 nm, respectively, by the spectrophotometer. The content of B vitamins group was determined as follows: Vitamin (μg/100g DW) = [(Absorbance of sample)/(Absorbance of standard) × (Dry weight of sample (μg) /(Dry weight of sample (g)] × 100.

The extraction and analysis of vitamin C was made as previously described by Law et al. [43]. Briefly, dried powdered seed sample (500 mg) was mixed with trichloroacetic acid (10% w/v) and then, 10 ml sodium hydroxide was added. To 200 μl of supernatant, 900 μ1 sodium phosphate monobasic buffer (200 mM, pH 7.4), and 200 μl DL-dithiothreitol (1.5 mM) were added. DL-Dithiothreitol was removed by adding 200 μl n-ethylmaleimide (0.5% w/v). To the reaction mixture, 1 ml trichloroacetic acid (10% w/v), 1 ml phosphoric acid (45% v/v), 1 ml 2,2'-bipyridyl (65 mM) in 70% (v/v) ethanol, and 500 μl iron (III) chloride (3% w/v) were added. The absorbance was read at 265 nm using a spectrophotometer (Bio-Tek Instruments, Inc., USA). Ascorbic acid (10–100 μg) was used as the standard.

α-Tocopherol content was determined according to the procedure detailed by Pant et al. [28]. In summary, 100 mg of seed powder was mixed with 2 ml petroleum ether and ethanol (1.6:2 v/v) and homogenized. The extract was centrifuged at 10,000 rpm for 20 min for 20 min. The supernatant was separated, and 40 μl of 2,2'-bipyridyl (2%, dissolved in ethanol) was added to 200 μl of the extract. The mixture was diluted with 800 µl of distilled water. Absorbance was read at 290 nm using a spectrophotometer. α-Tocopherol (10–50 μg/ml) was used as a standard.

## Proximate composition analysis

The samples were analyzed for ash, fat, protein, and fiber by the AOAC method [44]. Ash content was measured by heating the samples in a muffle oven (KSL-500X-71, MTI corporation, USA). The Soxhlet (SOX406, HANON Systems, South Korea) method was used for fat determination. Protein was estimated by the micro-Kjeldahl (K1100, HANON Systems, South Korea) method. Protein content was calculated by multiplying nitrogen content by a factor of 6.25. Crude fiber was obtained after sample digestion with boiling diluted acid and alkali. Carbohydrate content was determined by subtracting the sum of the weights of ash, fat, protein, and fiber from the total dry matter. Energy value was computed as follows: energy value (kcal/100 g) = 9 × (g of fat) + 4 × (g of protein + g of carbohydrates) [45].

## Determination of mineral composition

The concentrations of elements were determined after digestion [46], with pure nitric acid and hydrogen peroxide at 140 °C for 3 h and pressure dissolution by inductively coupled plasma mass spectrometry ICP-MS Agilent 7500A series (Agilent Technologies, Palo Alto, CA, USA). The operating parameters of the ICP-MS instrument are as follows: Rf power: 1100 w, Sampling depth: 2.5 mm, Nebulizes flow rate: 1.06 l/min., Plasma gas flow rate: 15 l/min., Auxiliary gas flow: 1.2 l/min., Helium flow rate: 5.0 ml/min., Fog chamber temperature: 2 °C, Sampling rate: 0.5 l/min., Signal measurement: Peak Hopping. Standard reference materials from the National Institute of Standards and Technology (NIST; Gaithersburg, MD, USA) were used to validate the analytical procedure.

## Extraction and determination of mucilage

Evaluation of mucilage was carried out as described by Verma et al. [32] and Singer et al. [47]. The mixture of seed and distilled water (1:40 w/v) was homogenized. Ethanol 96% (1:1 v/v) was used for mucilage deposition due to its effectiveness in dissolving alcohol-soluble components. The extracts were frozen and dried under vacuum for 48 h in a lyophilizer (CHRiST, alpha 1–2 Id plus, Germany). One gram of mucilage was added to 25 ml of distilled water in a cylinder. The swelling index was determined by measuring the volume occupied by mucilage. The isolated mucilage was characterized for organoleptic properties such as color and texture.

## Phytochemical analysis

### Fatty acid analysis

According to the AOAC procedures (AOAC 920.39), oil content was determined by Soxhlet extraction using *n*-hexane for 6 h. The seed oil compositions were measured using fatty acid methyl esters according to Milinsk et al. [48] with boron trifluoride due to an increase in volatility, thermal stability, and improvement of peak shape and separation behavior. The *n*-hexane extract was measured by GC–FID (Agilent Technologies, 7890A, USA). The GC–MS was installed with a universal column (HP5; 30 m 9 0.325 mm 9 0.25 lm; Agilent J&W GC column) with an autosampler. Helium was used as carrier gas at a flow rate of 1.2 ml/min with a split ratio of 1:100. The initial column temperature was maintained at 150 °C for 3 min and then increased to 240 °C at 3 °C/min with a 20 min hold time. Each sample was analyzed in three technical replicates. Determination and identification of fatty acids were used in the reference samples received from the NU-CHEK-PREP company (Code: GLC-462) (USA).

## Extraction and HPLC‒PDA determination of diosgenin, trigonelline, and phenolic compounds

Sample preparation for extraction of diosgenin was performed followed by Herrera et al. method [49]. Briefly, samples were extracted by sonication with methanol 1:10 (w/v) because of its polarity (Elma, S120H, Germany) for 30 min at 25 °C. Extracts centrifuged at 4,400 rpm for 10 min. Supernatants were defatted by the addition of the same volume of *n*-hexane. The methanolic phase was concentrated in a rotary (Heidolph Instruments GmbH, Schwabach Germany) at 35 °C. An equal volume of water-saturated *n*-butanol was added to the dry extract (50 mg/ml) and centrifuged at 4,400 for 10 min. The supernatant evaporated in a rotary at 35 °C. The dried extract was solved in 3 ml acetonitrile, and then filtered (0.22 μm).

Extraction of trigonelline was based on Campa et al. [50] with minor modifications. About 50 mg of the seed mixed with 25 ml distilled water and 50 mg of magnesium oxide. The mixture was placed in an autoclave at 105 °C for 20 min. The extract was filtered through a filter before analysis.

Phenolic compound extraction was carried out as described previously with modifications [33]. Initially, dried powdered seed sample (100 mg) was ultrasonically extracted with 10 ml methanol-DMSO (50:50 v/v,) for 30 min and centrifuged at 4,400 rpm for 5 min. The supernatants were dried in a rotary and dissolved in methanol with a final concentration of 1 ml.

The compounds analysis was carried out using a high-performance liquid chromatography-photodiode array (HPLC–PDA), with a Waters 2695 separations module equipped with a C_8_ column (50 × 2 mm, 3 μm) and a UV detector (Waters 2487). Water and acetonitrile (10:90 v/v) were used as mobile phases for diosgenin and trigonelline. The mobile phase for phenolic compounds was methanol with 1% formic acid. The flow rate was 0.5 ml/min. The detection was achieved using UV detector calibration curves drawn with regular diosgenin and trigonelline solutions at 210 and 263 nm, respectively.

## Quantification of total carotenoids, saponins, and tannins

Total carotenoid content was measured according to Pant et al. [28]. To determine carotenoid level, dried powdered seed sample (100 mg) was mixed with 5 ml acetone (80%) due to its fat solubility. The extract was partitioned with diethyl ether (5 ml). The dry extract was dissolved in ethanol (5 ml) and treated with potassium hydroxide (KOH) (60%). The extract was boiled for 10 min and then partitioned with diethyl ether. The dry extract was dissolved in 5 ml ethanol. The absorbance was calculated at wavelengths of 470 nm, using a spectrophotometer (Shimadzu double beam UV–Visible spectrophotometer-1800, Japan).

Total saponin content in fenugreek seed was determined following the methods of Akbari et al. [26], but slightly modified. Concisely, each dried powdered seed sample (500 mg) was extracted with 5 ml ethanol (64%), using a microwave-assisted extraction method (Milestone ETHOS UP, Italy) under 3 min irradiation time, 572 W microwave power. Then, 50 μl of the extract was mixed with 200 ml methanol, 100 μl vanillin/ethanol (10:90 w/v), and 300 μl sulfuric acid (70%). Absorbance was recorded at 544 using a spectrophotometer. Diosgenin (100‒500 mg/ml) was used as standard. Total saponin content was determined as follows: [the volume of extraction solvent (ml) × the concentration measured from diosgenin standard curve (mg/ml)]/the dry weight of the sample (g).

Total tannin content was determined according to Abdouli et al. [30, 31] with minor modifications. The seed (100 mg) was mixed with 5 ml diethyl ether containing 1% acetic acid. The mixture was centrifuged at 2,000 rpm for 10 min. Then, the supernatant was removed. Re-extraction was carried out with 5 ml of acetone (70%) and shocking for 60 min.

Total tannin was determined as the difference in total phenol content (TPC) based on the Folin-Ciocalteu method before and after the treatment with polyethylene glycol.

## Measurement of bitterness value

The bitterness value was performed as described previously [31, 51]. The stock solution of quinine hydrochloride contains 0.01 mg/ml. One g of plant material was extracted with 1000 ml of drinking water. The bitterness value was measured as follows formula [51]: Bitterness Value (unit/g) = [2000 × Quantity of quinine hydrochloride with the lowest bitter concentration (mg)]/[Concentration of the stock solution (mg/ml) × volume of stock solution with the lowest bitter concentration (ml)].

## Determination of total phenolic content (TPC) and total flavonoid content (TFC)

Total phenolic content (TPC) was measured according to the procedure detailed by Singleton et al. [52], with Folin-Ciocalteu reagent. The TFC was determined according to the method of Chang et al. [53] with aluminium chloride. Absorbance was taken at 765 nm and 510 nm for TPC and TFC, respectively.

## Antioxidant activity analysis

### DPPH scavenging activity

Antioxidant activity by the DPPH method was evaluated according to the methods described by Akhlaghi and Najafpour-Darzi [54]. The reduction of the DPPH radical was measured in a spectrophotometer at 515 nm. Butylated hydroxytol (BHT) was used as the control. The IC_50_ values were calculated as the following equation: DPPH scavenging effect (%) = (Abs _control_ ‒ Abs _sample_/Abs _control_) × 100. The IC_50_ value is defined as the concentration of substrate required to bring 50% scavenging activity of the DPPH radicals.

## Ferric reducing power

The methodology described by Benzie and Strain [55]. Absorbance was read at 593 nm by using a spectrophotometer. Different concentrations of iron sulfate (FeSO4 solution) were used for the calibration curve. The standard curve was constructed using FeSO_4_ solution (0.5–10 mg/ml).

## Data analysis

All the assays in the study were performed in triplicate. The data were analyzed using SPSS software version 21.0 (SPSS Inc., Chicago, IL, USA). One-way analysis of variance was used to test for significant differences. A post-hoc test was run using Duncan’s test at *p* < 0.05. All the experiments were conducted in triplicate, and the means and standard deviations were calculated in all tests. The cluster analysis was performed using Euclidean distance coefficient by Ward’s method. The Origin Lab software version 2021 was applied to draw the heat map and bi-plot.

## Results and discussion

### Vitamin content

**Fig. 2:**
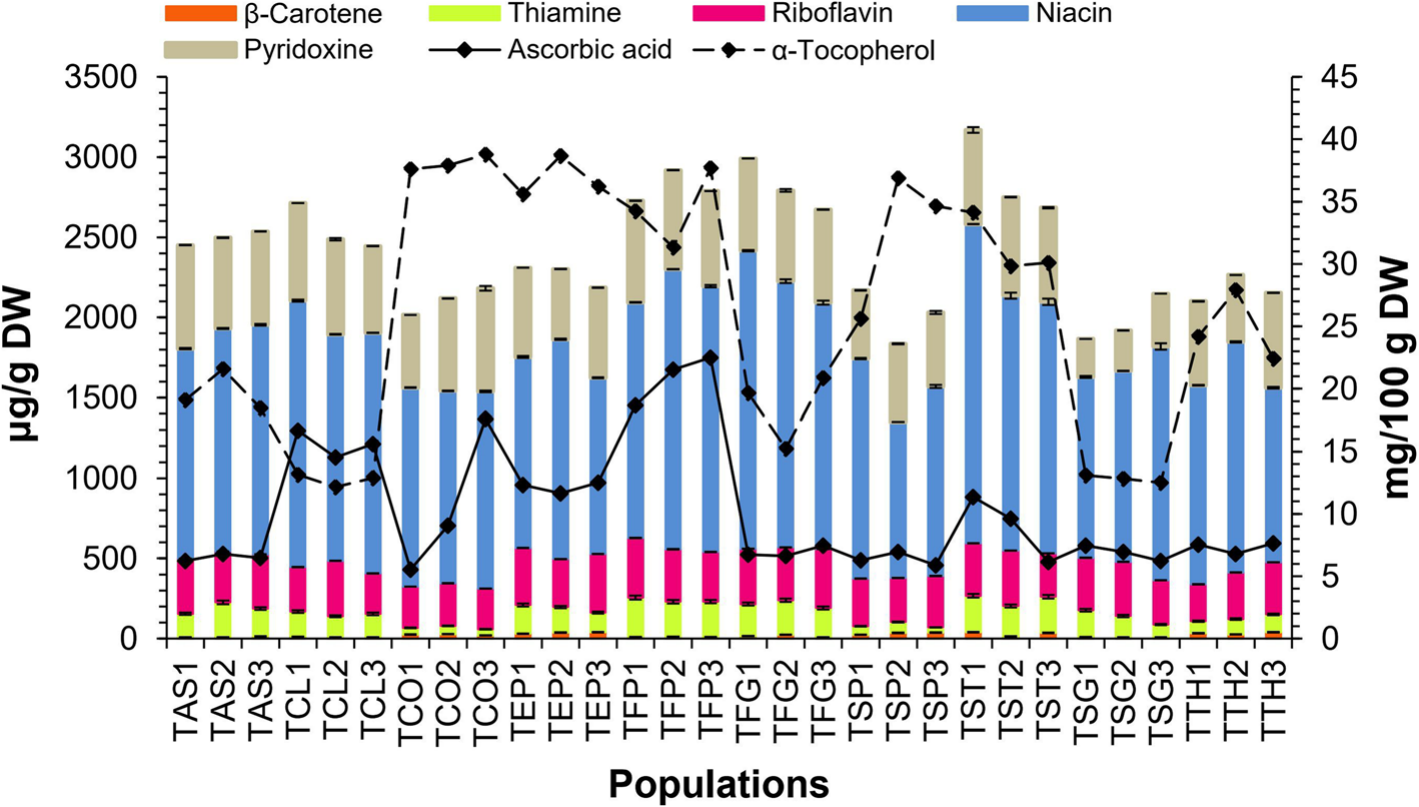
Histogram of vitamin content for thirty populations of ten *Trigonella* species

The results showed that there is a significant difference (*p* < 0.05) between the species and populations in terms of vitamin content. The level of vitamins (β-carotene, B group, C, and α–tocopherol) in the studied species and populations are shown in Fig. 2. The content of β-carotene was ranged from 8.19 ± 0.09 to 39.81 ± 1.90 μg/g DW. Among the studied seed samples, TTH3 and TCl3 had the highest and lowest β-carotene content, respectively. Thiamine content was found in the range of 32.64 ± 0.43 to 245.21 ± 3.15 μg/g DW. TFP1, TFP2, TFP3, TST1, TST2, TST3, and TAS2 had the highest content, while the lowest content was observed in TCO1, TCO2, TCO3, TSP1, TSP2, and TSP3. Riboflavin content varied from 231.11 ± 1.33 to 372.81 ± 1.98 μg/g DW. Among B group vitamins, niacin had the highest level that ranged from 972.42 ± 2.93 to 1987.54 ± 11.68 μg/g DW in the studied samples. TST1 contained the highest niacin level in the seeds. The lowest niacin content was observed in TSP2. Pyridoxine was ranged from 237.85 ± 3.25 to 645.64 ± 2.18 μg/g DW. Among the measured vitamins of the seed, α-tocopherol (vitamin E) and ascorbic acid (vitamin C) had the highest levels. α-Tocopherol content was ranged from 12.16 ± 0.10 to 38.78 ± 0.67 mg/100 g DW. The highest and lowest level of α-tocopherol belonged to the seeds of TCO3 and TCL2, respectively. The highest content of vitamin C was measured in TFP1, TFP2, and TFP3 (18.67 ± 0.85‒22.48 ± 0.60 mg/100 g DW), while the lowest level was observed in TCO1 (5.53 ± 0.05 mg/100 g DW).

Pant et al. [28] reported the content of α-tocopherol in the seeds of 25 genotypes of *T. foenum-graecum* from India in the range of 1 to 43 mg/100 g. In another study, α-tocopherol content of seed oil of *T. strangulata* Boiss. from Turkey was reported as 188.77 mg/100 g oil [37]. In the present study, α-tocopherol content for three populations of *T. strangulata* was found to be 12.50 ± 0.23 and 13.08 ± 0.64 mg/100 g DW. It has been reported that the seeds of *T. foenum-graecum* contain 96 μg/100 g β-carotene, 43 mg/100 g ascorbic acid, 340 μg/100 g thiamine, 290 μg/100 g riboflavin, and 1.1 mg/100 g niacin [29, 56] which is in agreement with our obtained results.

Out of thirteen vitamins that are necessary for the body, eight of them are in B group vitamins, which shows their significant role in human health and nutrition. In addition, fenugreek seeds contain a substantial amount of vitamins thiamine, riboflavin, and niacin [57]. Due to the lack of vitamins in all age groups, the need for new food sources has recently increased. Therefore, increasing the vitamin content in new foods, bread fortification with vitamin supplements, and enrichment of animal diets have attracted the attention of many countries such as the United States and Canada [58, 59]. In the present study, populations of *T. stellata*, *T. filipes* Boiss., *T. elliptica* Boiss., and *T. foenum-graecum* are introduced as adequate species of fenugreeks rich in group B vitamins, ascorbic acid, and α-tocopherol that can be interestingly considered for this purpose.

## Proximate composition

**Fig. 3:**
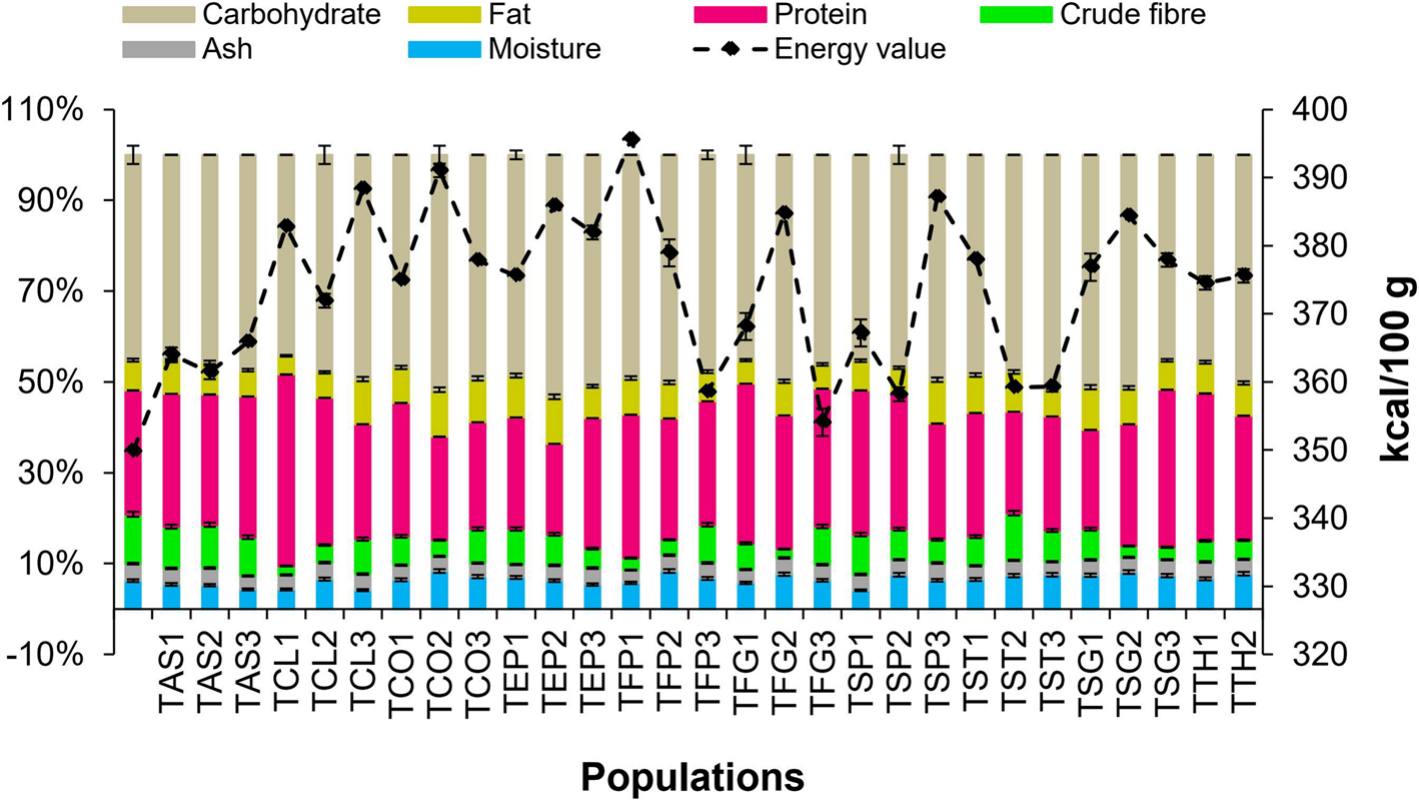
Histogram of proximate composition for thirty populations of ten *Trigonella* species

A significant difference (*p* < 0.05) was observed between the Iranian *Trigonella* species and their populations regarding proximate composition. Proximate composition and energy content are shown in Fig. 3. Seed moisture content varied from 4.10 ± 0.07% in TSP2 to 8.43 ± 0.56% in TCO3. The highest amount of ash was measured in TST1 (3.79 ± 0.17%). A wide difference in the amount of crude fiber was also observed in the studied samples (1.97 ± 0.10‒10.86 ± 0.44%). The fat content ranged from 4.12 ± 0.32% to 10.35 ± 0.77%. The highest fat content belonged to the TEP3 and TCO3.

Carbohydrate content was obtained in the range of 44.25 ± 0.75% to 53.24 ± 1.18%. The highest amount of protein was found in TCL2 (42.17 ± 0.74%), followed by TFG2 (35.20 ± 0.28%), and TTH1 (34.73 ± 0.31%). The highest and lowest amount of energy (kcal/100 g) was obtained in TFP2 (395.57% ± 3.06%) and TAS1 (349.85% ± 1.34%), respectively.

Although it has been reported the seeds of the cultivated fenugreek contain a significant amount of fat and protein, interestingly in the present study, some studied wild populations contained more which increases its nutritional value.

In previous studies, the carbohydrate content of the plant seeds had the highest percentage among proximate composition. Similar values of moisture, ash, crude fiber, and carbohydrates have also been reported in the seeds of *T. foenum-graecum* from Egypt and Iran [60, 61]. The fat content in seeds of *T. foenum-graecum* originating from four different countries was previously reported as 5.06 ± 0.61% to 6.33 ± 0.48% [62]. A wide range of protein content (4.4 to 28%) has been reported in fenugreek seeds so far [56, 63]. The results showed that the studied fenugreeks are a potential source of protein, fat, and carbohydrates that can be interestingly used in the food industries. In addition, it has been claimed that the proteins in fenugreeks have better quality compared to other vegetable proteins [23]. In a study, by changing the processing methods of fenugreek seeds, including sprouting and roasting, higher amounts of protein content were obtained, which can be useful for producing coffee-like drinks as well as microgreens with high protein content [64].

The increase in protein content in the germinated plant seeds can be due to the reduction of nitrate to protein or ammonium and enzymatic synthesis of protein [65, 66]. Optimization of protein extraction from fenugreek seeds resulted higher amounts than commercial soybean protein [61]. Data on the proximate composition of *Trigonella* wild species are not available in the literature. Values of protein and carbohydrate, fat, and crude fiber content in the present study widely varied among *T. foenum-graecum* in agreement with the variability of data reported. However, values of the protein, fat, and carbohydrate content in some of the studied wild species were higher than those reported for cultivated species of fenugreek. Considering that the amount of fenugreek seed protein in some populations of the studied wild species, including TCL2 (42.17 ± 0.74%), had significant values compared to the populations of its cultivated species (*T. foenum-graecum*) (26.71 ± 0.19–35.20 ± 0.28%), the fenugreek seed of some wild species can be proposed as an excellent source for improving the value of food formulations.

## Mineral composition

**Fig. 4:**
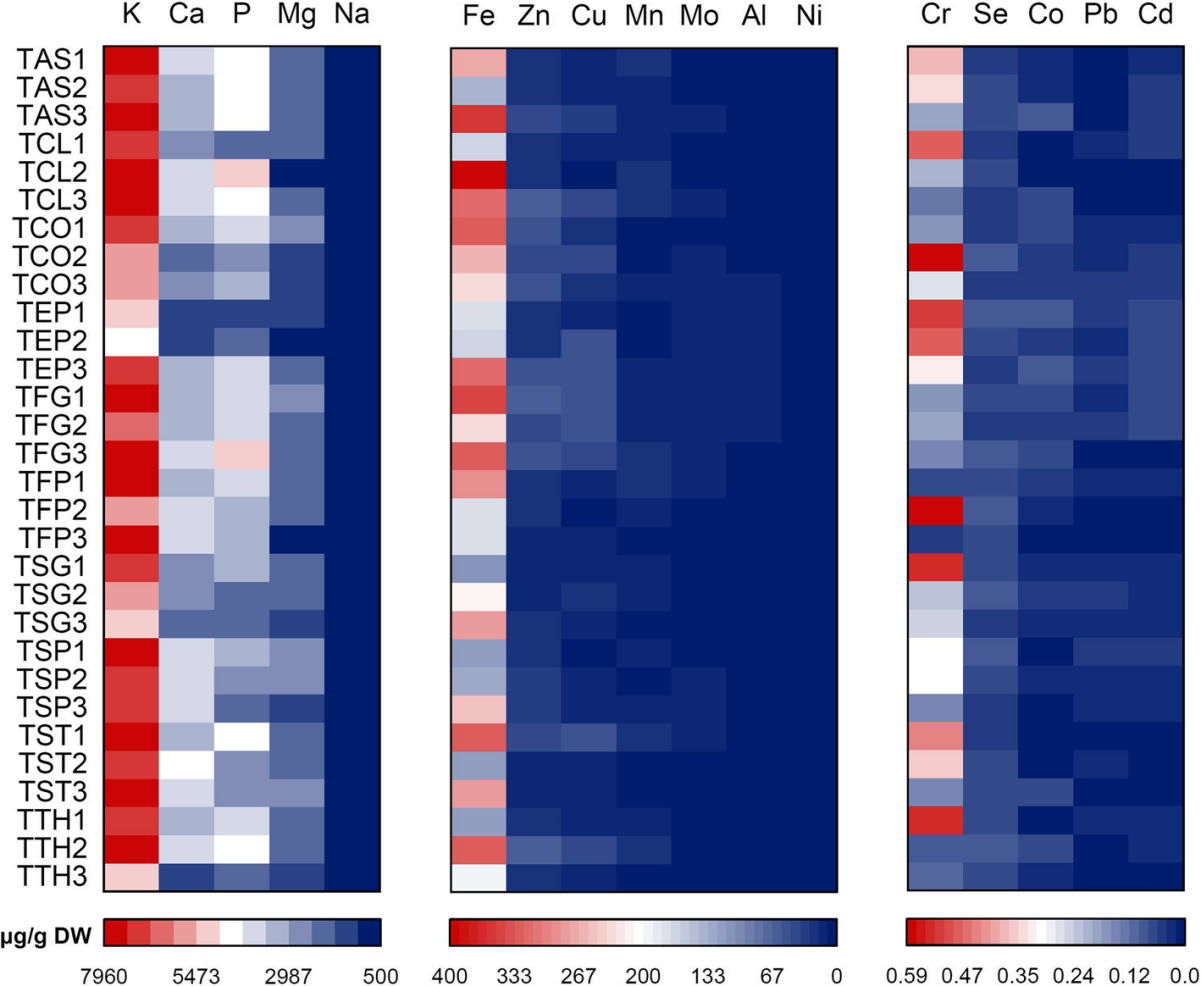
Comparisons of the levels of elements among the studied *Trigonella* species and their populations

A significant difference (*p* < 0.05) was observed between the species and populations regarding the studied elements. The results of the element content are shown in Fig. 4. The content of five macro-elements including potassium, calcium, magnesium, phosphorus, and sodium was measured as 4554.96 ± 7.08‒7942.89 ± 8.32, 1350.15 ± 2.26‒4325.63 ± 1.97, 744.58 ± 1.15‒2909.45 ± 4.89, 1156.63 ± 5.01‒4981.64 ± 4.40, and 502.64 ± 1.19‒780.52 ± 2.05 μg/g DW, respectively. The population of TFG1, followed by TST2, TCO1, TCL2, and TSG1, contained the highest value of macro-elements mentioned above. Among the micro-elements, iron had the highest content (112.96 ± 1.27‒398.75 ± 2.55 µg/g DW). The value of other elements were zinc, copper, manganese, molybdenum, aluminum, nickel, chromium, selenium, cobalt, cadmium, and lead in descending order.

In a study, potassium, manganese, phosphorus, copper, calcium, sodium, iron, and zinc content in the seeds of *T. foenum-graecum* from Sudan were reported as 1306, 1550, 415, 331, 158, 49, 22, and 10 mg/100 g, respectively [67]. In another study, it was shown that the seeds of thirteen cultivars of *T. foenum-graecum* from Turkey were rich in calcium (2341 µg/g) and magnesium (1372 µg/g) [68]. They have also been reported that there is a variation among the cultivars studied in terms of nickel, copper, cobalt, chromium, cadmium, aluminum and lead levels. Pandey and Awasthi [64], have reported that the seeds of *T. foenum-graecum* from India contained 544.5, 70.5, 11.6, and 5.7 mg/100 g of phosphorus, calcium, iron, and zinc, respectively. Variations in the content of different elements including calcium, chromium, manganese, zinc, copper, magnesium, selenium, aluminum, and lead in the seeds of ten fenugreek species from Turkey have also been reported [38]. Among the twenty-five *T. foenum-graecum* genotypes studied from India, the highest content of iron, calcium, manganese, copper, and zinc was 781.2, 25.65, 5.82, and 26.28 μg/g DW, respectively [28].

## Mucilage data

The content and characteristics of mucilage including pH, swelling index, and mucilage color, are given in Table 1. A significant difference (*p* < 0.05) was observed among the studied species and populations in the mentioned parameters.

**Table 1:** Physicochemical characterization of seed mucilage among the populations of *Trigonella* species

Populations	Mucilage content (%)	Mucilage pH	Swelling index (%)	Color
TAS1	13.16 ± 1.29^b^	6.40 ± 0.86^de^	84.89 ± 1.18^de^	Light yellow
TAS2	14.25 ± 1.43^b^	6.59 ± 0.34^cd^	84.33 ± 1.69^de^	Light brown
TAS3	13.99 ± 1.08^b^	6.42 ± 0.58^de^	80.34 ± 1.38^f^	Light yellow
TCL1	16.42 ± 0.89^ab^	7.20 ± 0.14^a^	88.64 ± 1.70^abc^	Light brown
TCL2	14.39 ± 0.75^b^	7.12 ± 0.28^a^	89.13 ± 1.65^abc^	Light brown
TCL3	14.94 ± 1.00^b^	7.10 ± 0.91^a^	88.50 ± 1.36^abc^	Light brown
TCO1	14.55 ± 1.56^b^	6.54 ± 0.08^cde^	82.12 ± 0.58^ef^	Light brown
TCO2	13.98 ± 1.38^b^	6.71 ± 0.77^bc^	85.34 ± 1.84^cd^	Yellow
TCO3	11.25 ± 1.15^b^	6.65 ± 0.59^cd^	76.80 ± 1.62^f^	Yellow
TEP1	19.66 ± 0.79^a^	6.72 ± 0.16^bc^	89.20 ± 1.27^abc^	Light green
TEP2	20.17 ± 1.06^a^	6.67 ± 0.47^cd^	84.99 ± 1.31^de^	Light green
TEP3	21.31 ± 1.18^a^	6.85 ± 0.26^ab^	85.33 ± 1.40^cd^	Light brown
TFP1	14.31 ± 0.99^b^	6.47 ± 0.75^de^	92.05 ± 1.53^a^	Light yellow
TFP2	15.65 ± 1.31^b^	6.28 ± 0.98^e^	91.24 ± 1.18^a^	Light brown
TFP3	15.79 ± 1.44^b^	6.31 ± 0.55^e^	90.00 ± 1.48^ab^	Light green
TFG1	14.04 ± 1.54^b^	7.00 ± 0.17^a^	90.25 ± 0.89^ab^	Light yellow
TFG2	13.77 ± 1.24^b^	7.15 ± 0.45^a^	90.14 ± 0.95^ab^	Light yellow
TFG3	16.56 ± 1.40^ab^	7.00 ± 0.66^b^	89.78 ± 1.34^abc^	Light yellow
TSP1	19.41 ± 1.26^a^	6.54 ± 0.47^cde^	87.20 ± 1.57^bcd^	Light green
TSP2	19.79 ± 1.13^a^	6.20 ± 0.22^e^	81.55 ± 1.50^ef^	Light yellow
TSP3	16.54 ± 0.35^ab^	6.87 ± 0.56^ab^	85.19 ± 1.24^cd^	Light green
TST1	15.62 ± 1.17^b^	6.49 ± 0.27^de^	90.36 ± 1.41^ab^	Light yellow
TST2	15.45 ± 0.56^b^	6.45 ± 0.19^de^	90.27 ± 1.78^ab^	Light brown
TST3	15.40 ± 0.73^b^	6.50 ± 0.42^cde^	89.95 ± 1.05^abc^	Light yellow
TSG1	16.06 ± 1.21^ab^	6.88 ± 0.12^ab^	85.19 ± 1.67^cd^	Light green
TSG2	19.63 ± 0.47^a^	6.93 ± 0.04^ab^	84.32 ± 1.50^de^	Light green
TSG3	18.92 ± 0.78^a^	7.00 ± 0.98^a^	87.41 ± 1.69^bcd^	Light green
TTH1	13.23 ± 0.89^b^	6.53 ± 0.07^cde^	76.15 ± 1.09^f^	Light green
TTH2	14.37 ± 0.52^b^	6.64 ± 0.61^cd^	78.61 ± 1.67^f^	Light brown
TTH3	14.62 ± 0.98^b^	6.68 ± 0.54^cd^	80.23 ± 1.45^f^	Light brown

For a detailed description of the plant species code, cf. Fig. 1

The highest mucilage content (21.31 ± 1.18%) was obtained in TEP3 followed, by TEP2 (20.17 ± 1.06%). The mucilage content in other populations was in the range of 11.25 ± 1.15% to 19.79 ± 1.13%. The pH of mucilage was in the range of 6.20 ± 0.22 to 7.20 ± 0.14. The lowest and highest swelling indices were observed in TTH1 (76.15 ± 1.09%) and TFP1 (92.05 ± 1.53%), respectively. Organoleptic properties including mucilage color and texture, were also measured among the studied fenugreeks. The mucilage color was varied in light yellow, yellow, light brown, and light green. The mucilage texture of all studied samples was rough and irregular.

The differences in mucilage content and characteristics among the studied species and populations can be attributed to their distinct origins and genetic structures. In a study, the mucilage content of *T. foenum-graecum* seeds from Iran was reported to be 23.86% (w/w), while mucilage acidity (pH) was 6.23 [27]. In another study, the extraction and determination of mucilage characteristics of *T. foenum-graecum* seeds from India were done. The amount of seed mucilage, pH, and swelling index were 15% (w/w), 7.9, and 90%, respectively. Also, the color of isolated mucilage was light brown with a rough and irregular texture [32].

Mucilage, a group of natural compounds, is widely utilized in food processing due to its beneficial physical properties. In addition, it is used in pharmaceutical industries for its binding, thickening, stabilizing, and humidifying effects [69]. Mucilage possesses valuable properties such as stabilization, emulsification, and suspension, making it a popular ingredient in the food, pharmaceutical, and cosmetic industries [70]. Furthermore, mucilage is preferred over semi-synthetic and synthetic types due to its non-toxicity, lower cost, easy accessibility, emollient properties, and non-irritating nature [71]. Notably, fenugreek seeds are reported to contain a significant amount of mucilage [27]. Fenugreek seed mucilage is acknowledged as a pharmaceutical excipient, serving as a binder in tablet formulation, gelling agent in diazepam nasal gels, and disintegrant in fast-dissolving tablets of amlodipine besylate [72].

In terms of mucilage content, the populations of *T. elliptica*, *T. spruneriana* Boiss., and *T. strangulata* species had the highest values, so these species can be recommended for further exploitation in the cultivation and breeding programs to cover food and pharmaceutical demands.

## Oil content and fatty acids composition

The obtained results revealed significant differences among the species and populations in terms of all the studied phytochemical traits. The seed oil content was in the range of 4.12% ± 0.32% to 10.35% ± 0.77%. The highest value was observed in TEP2. Seven fatty acids were identified in the studied samples, which made up 97.14‒99.78% of the seed oil. Saturated and unsaturated fatty acids (USFA) ranged from 19.77 ± 0.11% to 75.17 ± 1.52% and from 23.72 ± 0.37% to 80.01 ± 1.13%, respectively. Monounsaturated fatty acids (MUFA) ranged from 4.32 ± 0.26% to 24.49 ± 0.07%, while polyunsaturated fatty acids (PUFA) varied from 19.34 ± 1.42% to 63.46 ± 1.21% (Table 2). The seed oil was in bright yellow, dark red, and dark green colors and had a strong smell of celery, which can be of interest to the perfume industry [21].

**Table 2:** Fatty acid profiles of the studied *Trigonella* species

**FA (%)**	**TAS1**	**TAS2**	**TAS3**	**TCL1**	**TCL2**	**TCL3**
Oil (% w/w)	6.65 ± 0.27^cd^	7.32 ± 0.43^c^	7.12 ± 0.30^c^	5.81 ± 0.45^cd^	4.12 ± 0.32^d^	5.64 ± 0.59^d^
C14:0	0.04 ± 0.005^b^	0.04 ± 0.001^b^	0.05 ± 0.002^b^	0.01 ± 0.002^b^	0.06 ± 0.007^b^	0.09 ± 0.005^b^
C16:0	16.93 ± 1.12^de^	25.03 ± 0.81^de^	33.73 ± 1.32^bc^	54.84 ± 1.79^b^	52.50 ± 1.58^b^	55.39 ± 15^a^
C18:0	12.31 ± 0.15^bc^	10.69 ± 0.11^bcd^	9.18 ± 0.07^bcd^	6.46 ± 0.03^cd^	7.26 ± 0.09^cd^	6.28 ± 0.08^d^
C18:1n9	12.73 ± 0.07^b^	11.05 ± 0.06^bc^	10.30 ± 0.22^bc^	6.68 ± 0.12^cd^	6.56 ± 0.14^cd^	6.58 ± 0.25^cd^
C18:2n6	33.47 ± 0.44^a^	30.78 ± 0.29^ab^	26.78 ± 0.53^b^	19.28 ± 0.76^c^	19.02 ± 0.52^c^	17.95 ± 0.99^c^
C18:3n6	0.66 ± 0.03^b^	0.68 ± 0.02^b^	‒	‒	‒	‒
C18:3n3	20.98 ± 0.26^bc^	18.95 ± 0.34^bcd^	18.50 ± 0.49^bcd^	10.69 ± 0.21^de^	11.76 ± 0.43^de^	11.56 ± 0.23^de^
Others	2.86 ± 0.05^a^	2.77 ± 0.03^a^	1.46 ± 0.02^ab^	2.03 ± 0.03^ab^	2.84 ± 0.06^a^	2.16 ± 0.01^ab^
SFA	29.29 ± 0.87^d^	35.76 ± 0.96^cd^	42.96 ± 0.25^c^	61.31 ± 1.01^b^	59.82 ± 1.19^b^	61.76 ± 1.22^b^
USFA	67.84 ± 1.26^abc^	61.47 ± 1.33^bc^	55.58 ± 1.65^cd^	36.65 ± 1.15^e^	37.34 ± 0.78^e^	36.08 ± 0.71^e^
MUFA	12.73 ± 0.07^b^	11.05 ± 0.06^bc^	10.30 ± 0.22^bc^	6.68 ± 0.12^de^	6.56 ± 0.14^de^	6.58 ± 0.25^de^
PUFA	55.11 ± 1.17^cd^	50.41 ± 1.04^c^	45.28 ± 0.79^c^	29.97 ± 1.14^cd^	30.77 ± 0.89^d^	29.51 ± 0.55^d^
**FA (%)**	**TCO1**	**TCO2**	**TCO3**	**TEP1**	**TEP2**	**TEP3**
Oil (% w/w)	9.98 ± 0.18^a^	7.82 ± 0.17^b^	10.34 ± 0.09^a^	9.64 ± 0.59^a^	9.22 ± 0.59^ab^	10.35 ± 0.77^a^
C14:0	0.04 ± 0.004^b^	0.03 ± 0.002^b^	0.05 ± 0.003^b^	0.11 ± 0.010^b^	0.07 ± 0.003^b^	0.09 ± 0.004^b^
C16:0	30.50 ± 1.02^cd^	32.42 ± 0.98^c^	29.99 ± 0.65^d^	67.81 ± 1.15^a^	62.92 ± 0.46^a^	70.46 ± 0.87^a^
C18:0	9.76 ± 0.05^bcd^	7.91 ± 0.15^cd^	11.42 ± 0.13^bc^	4.73 ± 0.05^d^	4.87 ± 0.42^d^	4.62 ± 0.19^d^
C18:1n9	8.49 ± 0.26^cd^	11.23 ± 0.24^bc^	9.86 ± 0.31^bcd^	5.86 ± 0.63^cd^	4.87 ± 0.04^d^	4.32 ± 0.26^d^
C18:2n6	30.63 ± 0.28^ab^	30.68 ± 0.32^ab^	31.91 ± 0.24^ab^	13.04 ± 0.19^c^	14.45 ± 0.11^c^	11.94 ± 0.12^c^
C18:3n6	‒	‒	‒	‒	1.43 ± 0.08^a^	‒
C18:3n3	19.93 ± 0.14^bcd^	17.39 ± 0.06^bcd^	16.20 ± 0.62^cd^	6.30 ± 0.10^e^	8.90 ± 0.04^de^	7.46 ± 0.06^e^
Others	0.65 ± 0.03^b^	0.34 ± 0.02^b^	0.56 ± 0.03^b^	2.14 ± 0.04^ab^	2.47 ± 0.02^a^	1.11 ± 0.06^ab^
SFA	40.30 ± 0.26^c^	40.36 ± 0.64^c^	41.46 ± 0.38^c^	72.66 ± 1.88^a^	67.87 ± 1.65^ab^	75.17 ± 1.52^a^
USFA	59.05 ± 0.54^c^	59.30 ± 0.65^c^	57.97 ± 1.50^c^	25.20 ± 1.22^f^	29.66 ± 1.13^e^	23.72 ± 0.37^f^
MUFA	8.49 ± 0.26^cd^	11.23 ± 0.24^bc^	9.86 ± 0.31^bcd^	5.86 ± 0.63^e^	4.87 ± 0.04^e^	4.32 ± 0.26^e^
PUFA	50.56 ± 0.67^a^	48.07 ± 1.00^b^	48.11 ± 2.04^a^	19.34 ± 1.42^a^	24.79 ± 0.56^ab^	19.40 ± 1.21^a^
**FA (%)**	**TFP1**	**TFP2**	**TFP3**	**TFG1**	**TFG2**	**TFG3**
Oil (% w/w)	7.03 ± 0.54^c^	8.09 ± 0.30^b^	7.97 ± 0.55^b^	6.53 ± 0.42^cd^	5.18 ± 0.85^d^	7.52 ± 0.43^bc^
C14:0	0.74 ± 0.043^a^	0.09 ± 0.001^b^	0.21 ± 0.013^ab^	0.06 ± 0.006^b^	0.07 ± 0.001^b^	0.46 ± 0.028^ab^
C16:0	15.87 ± 0.43^de^	13.06 ± 0.17^de^	18.95 ± 0.09^de^	13.81 ± 0.12^de^	32.57 ± 0.49^c^	30.20 ± 0.23^cd^
C18:0	8.87 ± 0.13^cd^	10.90 ± 0.01^bcd^	9.43 ± 0.15^bcd^	25.74 ± 0.11^a^	11.42 ± 0.34^bc^	11.30 ± 0.18^bc^
C18:1n9	9.98 ± 0.17^bcd^	12.70 ± 0.30^b^	10.73 ± 0.15^bc^	22.68 ± 0.04^a^	9.82 ± 0.09^bcd^	10.84 ± 0.24^bc^
C18:2n6	35.76 ± 0.38^a^	38.13 ± 0.33^a^	31.08 ± 0.46^ab^	0.01 ± 0.00^d^	27.55 ± 0.25^b^	30.51 ± 0.87^ab^
C18:3n6	0.98 ± 0.09^ab^	1.11 ± 0.04^a^	1.24 ± 0.04^a^	1.02 ± 0.01^ab^	‒	‒
C18:3n3	26.24 ± 0.18^b^	21.40 ± 0.15^bc^	25.60 ± 0.12^b^	33.92 ± 0.27^b^	15.72 ± 0.33^cd^	15.69 ± 0.23^cd^
Others	1.56 ± 0.04^ab^	2.59 ± 0.02^a^	2.76 ± 0.02^a^	2.76 ± 0.01^a^	2.85 ± 0.04^a^	‒
SFA	25.48 ± 0.34^d^	24.06 ± 0.60^d^	28.59 ± 0.23^d^	39.61 ± 0.78^c^	44.06 ± 0.50^c^	41.96 ± 0.63^c^
USFA	72.96 ± 0.26^ab^	73.35 ± 0.87^a^	68.65 ± 0.70^abc^	57.63 ± 1.02^c^	53.09 ± 0.94^cd^	58.04 ± 0.24^c^
MUFA	9.98 ± 0.17^bcd^	12.70 ± 0.30^b^	10.73 ± 0.15^bc^	22.68 ± 0.04^a^	9.82 ± 0.09^bcd^	10.84 ± 0.24^bc^
PUFA	62.98 ± 1.17^c^	60.65 ± 1.45^b^	57.92 ± 0.67^b^	34.95 ± 0.34^cd^	43.27 ± 0.98^d^	47.20 ± 0.79^bc^
**FA (%)**	**TSP1**	**TSP2**	**TSP3**	**TST1**	**TST2**	**TST3**
Oil (% w/w)	5.37 ± 0.78^d^	6.55 ± 0.74^cd^	5.69 ± 0.99^d^	9.65 ± 0.14^a^	8.31 ± 0.10^ab^	8.78 ± 0.25^ab^
C14:0	0.02 ± 0.004^b^	0.08 ± 0.004^b^	0.04 ± 0.007^b^	0.44 ± 0.056^ab^	0.01 ± 0.000^b^	0.05 ± 0.003^b^
C16:0	39.87 ± 1.35^bc^	44.89 ± 1.47^b^	42.30 ± 1.22^bc^	18.89 ± 0.53^de^	19.89 ± 0.76^de^	15.79 ± 0.17^de^
C18:0	8.45 ± 0.12^cd^	11.88 ± 0.16^bc^	9.74 ± 0.17^bcd^	0.44 ± 0.25^d^	11.63 ± 0.41^bc^	12.36 ± 0.37^ab^
C18:1n9	11.17 ± 0.20^bc^	7.79 ± 0.19^cd^	8.13 ± 0.05^cd^	16.55 ± 0.10^a^	10.87 ± 0.08^bc^	11.15 ± 0.01^bc^
C18:2n6	26.94 ± 0.63^b^	19.44 ± 0.35^c^	21.80 ± 0.53^bc^	39.11 ± 0.61^a^	31.10 ± 0.44^ab^	35.21 ± 0.26^a^
C18:3n6	0.41 ± 0.02^b^	0.68 ± 0.02^b^	0.50 ± 0.01^b^	1.03 ± 0.04^ab^	‒	0.68 ± 0.05^b^
C18:3n3	11.95 ± 0.09^de^	14.29 ± 0.06^cde^	15.66 ± 0.10^cd^	23.32 ± 0.16^b^	23.72 ± 0.73^b^	24.28 ± 0.16^b^
Others	1.19 ± 0.05^ab^	0.95 ± 0.02^b^	1.84 ± 0.03^ab^	0.22 ± 0.02^b^	2.78 ± 0.03^a^	0.48 ± 0.05^b^
SFA	48.34 ± 0.98^c^	56.85 ± 1.14^b^	52.08 ± 1.24^b^	19.77 ± 0.11^d^	31.53 ± 0.46^d^	28.20 ± 0.25^d^
USFA	50.47 ± 0.79^cd^	42.20 ± 0.42^d^	46.08 ± 0.25^d^	80.01 ± 1.13^a^	65.69 ± 1.51^abc^	71.33 ± 1.87^ab^
MUFA	11.17 ± 0.20^bc^	7.79 ± 0.19^de^	8.13 ± 0.05^cd^	16.55 ± 0.10^b^	10.87 ± 0.08^bc^	11.15 ± 0.01^bc^
PUFA	39.30 ± 1.56^d^	34.41 ± 1.31^cd^	37.95 ± 1.33^d^	63.46 ± 1.21^a^	54.81 ± 1.39^ab^	60.17 ± 1.45^ab^
**FA (%)**	**TSG1**	**TSG2**	**TSG3**	**TTH1**	**TTH2**	**TTH3**
Oil (% w/w)	5.71 ± 0.16^cd^	9.41 ± 0.09^ab^	7.98 ± 0.11^b^	6.45 ± 0.3^cd^	6.92 ± 0.92^cd^	7.23 ± 0.54^c^
C14:0	0.72 ± 0.020^a^	0.08 ± 0.001^b^	0.29 ± 0.011^ab^	0.03 ± 0.002^b^	0.04 ± 0.003^b^	0.05 ± 0.005^b^
C16:0	15.01 ± 0.30^de^	29.45 ± 0.44^d^	21.30 ± 0.25^de^	25.45 ± 0.71^de^	26.97 ± 0.90^d^	0.01 ± 0.00^e^
C18:0	10.85 ± 0.16^bcd^	10.76 ± 0.19^bcd^	15.13 ± 0.10^ab^	18.83 ± 0.05^a^	18.57 ± 0.13^a^	22.88 ± 0.01^a^
C18:1n9	12.28 ± 0.09^b^	8.82 ± 0.04^cd^	10.08 ± 0.06^bc^	18.94 ± 0.26^a^	19.56 ± 0.44^a^	24.49 ± 0.07^a^
C18:2n6	34.13 ± 0.55^a^	32.69 ± 0.21^a^	29.75 ± 0.38^b^	0.01 ± 0.00^d^	0.01 ± 0.00^d^	0.01 ± 0.00^d^
C18:3n6	0.80 ± 0.06^b^	0.03 ± 0.00^b^	0.09 ± 0.03^b^	1.22 ± 0.04^a^	1.46 ± 0.01^a^	1.56 ± 0.07^a^
C18:3n3	23.60 ± 0.24^b^	17.76 ± 0.40^bcd^	22.80 ± 0.13^b^	33.72 ± 0.27^b^	32.49 ± 0.64^b^	48.78 ± 0.39^a^
Others	2.62 ± 0.04^a^	0.41 ± 0.01^b^	0.56 ± 0.05^b^	1.80 ± 0.03^ab^	0.91 ± 0.02^b^	2.22 ± 0.08^ab^
SFA	26.57 ± 0.42^d^	40.29 ± 0.13^c^	36.72 ± 0.26^c^	44.32 ± 1.11^c^	45.58 ± 1.35^c^	22.94 ± 0.94^d^
USFA	70.81 ± 1.10^ab^	59.30 ± 1.18^c^	62.72 ± 0.57^bc^	53.88 ± 0.49^cd^	53.51 ± 0.73^cd^	74.84 ± 0.95^a^
MUFA	12.28 ± 0.09^b^	8.82 ± 0.04^cd^	10.08 ± 0.06^bc^	18.94 ± 0.26^ab^	19.56 ± 0.44^a^	24.49 ± 0.07^a^
PUFA	58.53 ± 1.24^cd^	50.48 ± 1.56^ab^	52.64 ± 1.21^b^	34.94 ± 0.87^cd^	33.95 ± 0.58^cd^	50.35 ± 0.72^c^

Each value is expressed as the mean ± SD (*n* = 3) of triplicate determinations

Means with different letters within a row are significantly different (*p* < 0.05)

*FA* fatty acids, *SFA* saturated fatty acids, *USFA* unsaturated fatty acids, *MUFA* monounsaturated fatty acids, *PUFA* polyunsaturated fatty acids

For a detailed description of the plant species code, cf. Fig. 1

TST1, TTH3, and TFP1 had the lowest rate of USFA, while TEP1, TEP2, and TEP3 had the highest values. The highest percentage of USFA was determined in TST1, TST2, and TST3. TEP1, TEP2, and TEP3 were the main saturated fatty acids in the studied samples. Palmitic acid (0.00 ± 0.00‒70.46 ± 0.87%) is the main saturated fatty acid. Linoleic acid (0.00 ± 0.00‒39.11% ± 0.61%) and linolenic acid (6.30 ± 0.10‒48.78 ± 0.39%) were also the main polyunsaturated fatty acids. γ-Linoleic acid was the lowest (0.00 ± 0.00‒1.56 ± 0.07%). The only MUFA was oleic acid, which had the highest values ​​in TTH1, TTH2, TTH3, and TFG1. Typical chromatograms of fatty acids are shown in Supplementary Fig. 1.

Studies have shown that linolenic acid is the predominant fatty acid in oily seeds of flowering plants [73]. Unsaturated fatty acids are considered beneficial fats because they can improve blood cholesterol levels, ease inflammation, and stabilize heart rhythms [74]. Today, researchers have discovered that fenugreek seeds contain 6–8% oil with a high percentage of USFA, making it suitable as a food supplement for edible oils [75]. Moreover, the seed oil extracted from fenugreek is highly beneficial in the pharmaceutical industry due to its antioxidant, anti-cancer, and anti-diabetic properties [76, 77].

The main fatty acids in fenugreek seeds from Turkey and India are polyunsaturated fatty acids, including linoleic acid, linolenic acid, and palmitic acid has been reported as the main saturated fatty acid so far [38, 78–80]. The saturation and unsaturation of fatty acids and their levels in plants are affected by environmental conditions such as temperature, rainfall and genotype [81].

In the present study, most of the studied species and populations contained USFA including linoleic acid and linolenic acid, which according to the same cultivation conditions, it can be concluded that the profile of fatty acids is attributed to the plant genotype and origins. Considering the importance of USFA including linoleic acid and linolenic acid for human heart health, cultivation of *Trigonella* species rich in these compounds for the consumption of their seeds is revealed. Results of this study suggest that farmers should grow the populations of *T. stellata* and *T. filipes* to produce the highest economic amount of oil and USFA for therapeutic and food purposes.

## Phenolic compounds

**Fig. 5:**
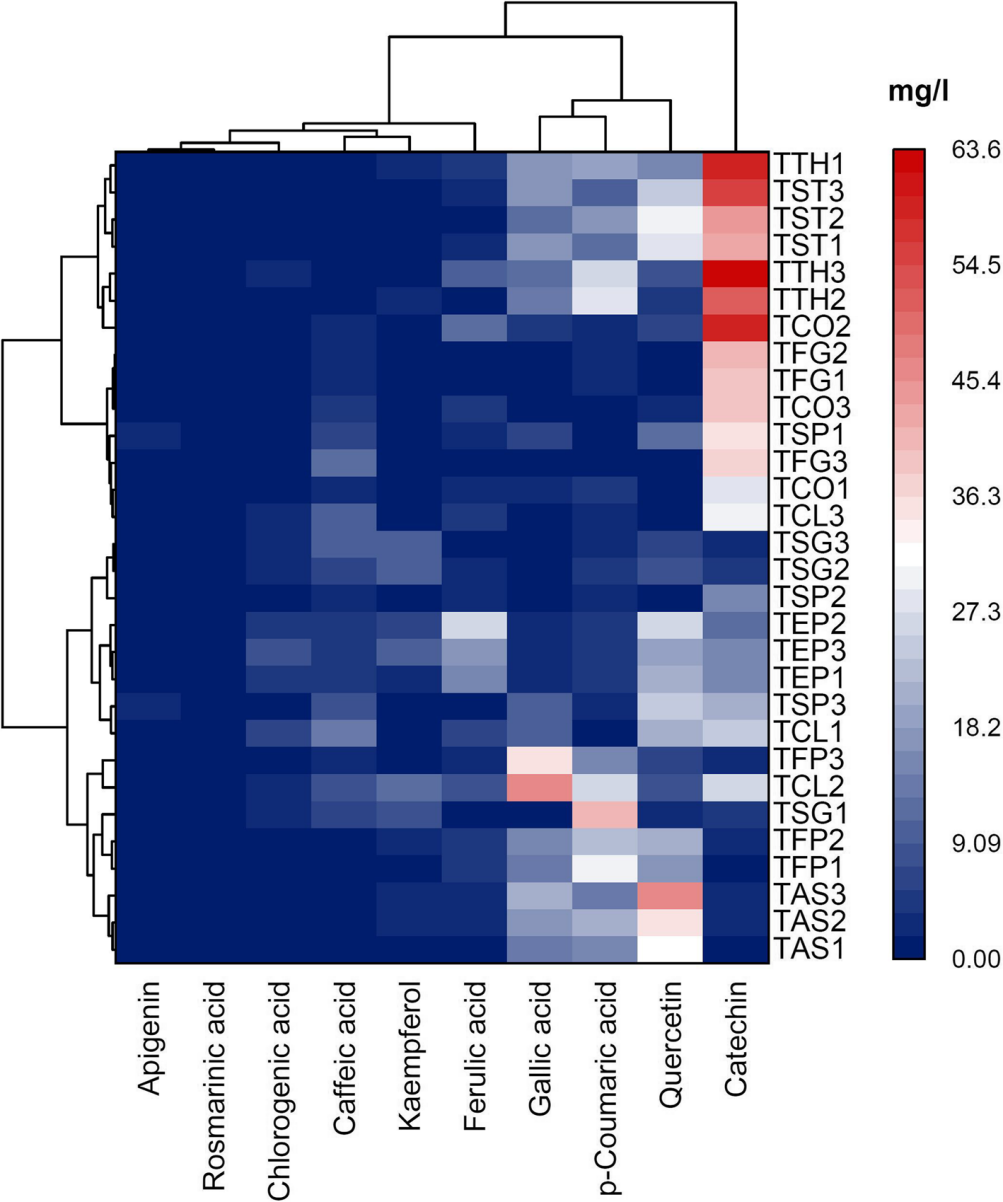
Content of major phenolic compounds identified among the thirty populations of ten *Trigonella* species

Fig. 5 shows the range of ten phenolic compounds found in the studied fenugreeks. As can be seen, catechin was the most abundant phenolic compound in the studied samples with the highest content found in TTH3 (1.67 ± 0.05‒63.50 ± 0.72 mg/l). The other measured phenolic compounds were gallic acid (0.00 ± 0.00‒46.96 ± 0.26), quercetin (0.44 ± 0.00‒45.54 ± 0.15), *p*-coumaric acid (0.57 ± 0.05‒40.65 ± 0.10), ferulic acid (0.00 ± 0.00‒26.00 ± 0.11), caffeic acid (0.40 ± 0.01‒13.55 ± 0.09), kaempferol (0.00 ± 0.00‒12.47 ± 0.01), and chlorogenic acid (0.00 ± 0.00‒7.33 ± 0.09). The content of apigenin (0.00 ± 0.00‒2.78 ± 0.05 mg/l) and rosmarinic acid (0.00 ± 0.00‒0.98 ± 0.02 mg/l) was negligible. The highest content of these phenolic compounds was determined in *T. teheranica* and *T. stellata*. Rosmarinic acid was not found in more than half of the studied samples.

Heatmap is a valuable tool for gaining insights from data visually and intuitively, allowing the researchers to make data-driven decisions and identify patterns or areas of interest within the data. Heatmap analysis classified the studied populations into two main groups based on the content of phenolic compounds (Fig. 5). The group *I* included the populations of *T. teheranica* (Bornm.) Grossh., *T. stellata*, *T. coerulescens* (M.Bieb.) Halacsy, *T. foenum-graecum*, TCL3, and TSP1 are characterized by a high content of catechin. Group *II* comprises the populations of *T. astroides* Fisch. & C.A.Mey., *T. filipes, T. strangulata, T. elliptica*, TSP2, TSP3, TCL1, and TCL2, were associated with the high amount of *p*-coumaric acid, quercetin, and gallic acid.

Belguith-Hadriche et al. [82] have reported the presence of the three flavonoid glycosides including kaempferol (3.20 ± 0.12 μg/mg), apigenin (5.11 ± 0.15 μg/mg), and naringenin (7.23 ± 0.09 μg/mg) from the ethyl acetate extract of fenugreek seeds from Tunisia. In another study, apigenin (19,555 ng/mg) and luteolin (725 ng/mg) were the most abundant phenolic compounds in *T. foenum-graecum* seeds from the UK [83]. Vitexin and isovitexin were also reported as the major phenolic compounds in fenugreek germinated seeds from India [84].

## Diosgenin and trigonelline content

The diosgenin and trigonelline contents of the studied samples are presented in Table 3. TST1, TST2, TST3, TFG1, TFG2, TFG3, TCO1, and TCO2 had the highest diosgenin content (9.06 ± 0.06‒11.03 ± 0.17 mg/g DW), while the lowest diosgenin content (2.52 ± 0.01 mg/g DW) was obtained in TFP2. Trigonelline content ranged from 0.68 ± 0.01 mg/g DW to 7.66 ± 0.16 mg/g DW. The highest and lowest trigonelline content was obtained in TST1 and TSP2, respectively.

**Table 3:** Variation in the phytochemical traits among the populations of ten *Trigonella* species

Populations	Diosgenin (mg/g DW)	Trigonelline (mg/g DW)	Total carotenoids (μg β-carotene/g DW)	Total saponins (mg DE/g DW)	Total tannins (mg/100 g DW)	Bitterness value (units × 10^3^/g)
TAS1	2.64 ± 0.03^d^	3.38 ± 0.09^cd^	19.63 ± 0.09^d^	29.66 ± 0.17^e^	394.63 ± 1.86^a^	0.96 ± 0.01^c^
TAS2	4.42 ± 0.02^d^	3.22 ± 0.05^cde^	18.45 ± 0.12^d^	21.32 ± 0.28^e^	378.85 ± 1.96^a^	1.78 ± 0.02^c^
TAS3	3.55 ± 0.04^d^	3.24 ± 0.01^cde^	31.23 ± 0.43^c^	28.54 ± 0.15^e^	369.96 ± 1.34^a^	0.98 ± 0.00^c^
TCL1	8.84 ± 0.05^b^	5.02 ± 0.05^ab^	28.51 ± 0.42^cd^	171.30 ± 1.14^ab^	265.82 ± 0.92^bc^	4.09 ± 0.03^a^
TCL2	5.43 ± 0.04^c^	3.77 ± 0.02^cd^	19.60 ± 0.10^d^	125.44 ± 0.95^cd^	218.47 ± 1.75^c^	2.48 ± 0.05^b^
TCL3	7.78 ± 0.01^d^	4.32 ± 0.04^abc^	16.84 ± 0.05^d^	159.58 ± 0.64^abc^	182.65 ± 0.88^d^	3.56 ± 0.01^a^
TCO1	9.26 ± 0.04^a^	2.23 ± 0.02^ef^	41.26 ± 0.21^bc^	168.11 ± 1.12^ab^	89.52 ± 0.65^d^	3.89 ± 0.06^a^
TCO2	9.78 ± 0.10^a^	2.27 ± 0.01^ef^	44.50 ± 0.46^bc^	207.64 ± 0.97^a^	98.08 ± 0.29^d^	4.58 ± 0.12^a^
TCO3	8.38 ± 0.00^b^	2.25 ± 0.00^ef^	39.90 ± 0.38^bc^	160.32 ± 0.86^abc^	207.96 ± 0.74^c^	3.14 ± 0.14^ab^
TEP1	7.16 ± 0.05^bc^	2.04 ± 0.00^ef^	49.23 ± 0.52^bc^	199.42 ± 0.56^a^	109.97 ± 1.36^d^	3.45 ± 0.06^a^
TEP2	6.61 ± 0.01^c^	2.12 ± 0.03^ef^	58.08 ± 0.18^ab^	146.38 ± 0.70^bcd^	72.43 ± 0.49^d^	3.56 ± 0.03^a^
TEP3	6.39 ± 0.03^c^	1.03 ± 0.01^f^	62.83 ± 0.73^a^	143.70 ± 0.19^bcd^	71.89 ± 0.95^d^	3.47 ± 0.07^a^
TFP1	2.63 ± 0.06^d^	4.25 ± 0.08^abc^	31.29 ± 0.29^c^	59.52 ± 0.44^e^	267.86 ± 1.51^bc^	0.95 ± 0.01^c^
TFP2	2.52 ± 0.01^d^	5.61 ± 0.01^a^	70.39 ± 0.68^a^	60.91 ± 0.21^e^	263.90 ± 1.60^bc^	0.97 ± 0.00^c^
TFP3	4.55 ± 0.02^d^	4.28 ± 0.03^abc^	17.83 ± 0.09^d^	145.46 ± 0.18^bcd^	216.34 ± 1.43^c^	2.26 ± 0.04^bc^
TFG1	9.25 ± 0.06^a^	6.78 ± 0.07^a^	25.12 ± 0.25^cd^	165.24 ± 0.78^ab^	180.31 ± 0.78^d^	4.23 ± 0.08^ab^
TFG2	9.33 ± 0.08^a^	5.33 ± 0.02^ab^	27.32 ± 0.18^cd^	178.52 ± 1.52^ab^	98.56 ± 0.49^d^	4.34 ± 0.05^a^
TFG3	10.45 ± 0.14^a^	4.26 ± 0.03^abc^	20.87 ± 0.11^cd^	200.67 ± 1.67^a^	79.15 ± 0.81^d^	4.19 ± 0.05^a^
TSP1	6.07 ± 0.02^c^	2.15 ± 0.05^ef^	65.86 ± 0.30^a^	156.88 ± 1.10^abc^	213.45 ± 1.09^c^	2.15 ± 0.00^c^
TSP2	5.95 ± 0.03^c^	0.68 ± 0.01^f^	35.96 ± 0.15^c^	145.32 ± 0.99^bcd^	211.98 ± 1.24^c^	2.12 ± 0.01^c^
TSP3	4.09 ± 0.06^d^	1.93 ± 0.02^f^	54.59 ± 0.66^ab^	130.06 ± 0.74^bcd^	200.86 ± 1.20^d^	2.56 ± 0.01^b^
TST1	9.06 ± 0.06^ab^	7.66 ± 0.16^a^	15.43 ± 0.12^d^	187.37 ± 1.01^ab^	175.35 ± 1.59^d^	4.45 ± 0.09^a^
TST2	9.15 ± 0.09^ab^	5.17 ± 0.01^ab^	15.11 ± 0.18^d^	208.86 ± 1.27^a^	81.37 ± 0.89^d^	4.13 ± 0.13^a^
TST3	11.03 ± 0.17^a^	4.95 ± 0.03^ab^	15.23 ± 0.06^d^	211.58 ± 1.54^a^	78.45 ± 0.61^d^	4.45 ± 0.11^a^
TSG1	5.79 ± 0.03^c^	3.30 ± 0.09^cde^	74.21 ± 0.91^a^	147.45 ± 0.25^bcd^	217.32 ± 1.38^c^	2.90 ± 0.02^b^
TSG2	2.71 ± 0.01^d^	2.85 ± 0.06^ef^	73.16 ± 0.49^a^	73.21 ± 0.69^d^	357.75 ± 1.70^b^	1.00 ± 0.03^c^
TSG3	2.92 ± 0.02^d^	3.19 ± 0.05^def^	76.45 ± 0.76^a^	76.53 ± 0.45^d^	326.40 ± 1.05^b^	1.01 ± 0.05^c^
TTH1	3.24 ± 0.02^d^	2.91 ± 0.04^ef^	52.27 ± 0.35^b^	126.41 ± 0.50^bcd^	257.47 ± 1.92^bc^	0.98 ± 0.09^c^
TTH2	5.95 ± 0.04^c^	4.25 ± 0.07^abc^	54.35 ± 0.42^ab^	127.55 ± 0.91^bcd^	245.55 ± 1.36^bc^	2.45 ± 0.06^b^
TTH3	4.41 ± 0.01^d^	4.84 ± 0.02^abc^	77.29 ± 0.99^a^	120.83 ± 0.32^cd^	209.18 ± 1.15^c^	2.45 ± 0.05^b^

Each value is expressed as the mean ± SD (*n* = 3) of triplicate determinations

Means with different letters within a column are significantly different (*p* < 0.05)

For a detailed description of the plant species code, cf. Fig. 1

In a previous study, the content of diosgenin in the seeds of ten *T. foenum-graecum* populations from Canada has been ranged from 3.29 to 6.43 mg/g [85]. In another study, diosgenin content in seed of *T. foenum-graecum* from India was also found in the range of 0.53 to 0.65% (w/w) [86].

Among fifteen *Trigonella* species from Australia [87], *T. foenum-graecum* seed had a higher diosgenin content (5.09 ± 0.35 mg/g). *Trigonella anguina* Delile, *T. spicata* Sm., and *T. caerulea* (L.) Ser. also contained 3.72 ± 0.17, 1.39 ± 0.10, and 2.46 ± 0.24 mg/g, respectively. The seeds of *T. calliceras* Fisch., *T. filipes*, and *T. coerulescens* lacked diosgenin, while our obtained results showed that the content of diosgenin ranged from 2.52 ± 0.01 to 9.87 ± 0.10 mg/g DW in the studied species mentioned above. In a comparative study from Turkey [38], the highest diosgenin content (0.52 ± 0.01 mg/g) was reported in *T. cilicica* Hub.-Mor. Diosgenin content in *T. spruneriana* and *T. filipes* Boiss. seed were less than our samples. In another study on thirteen genotypes of *T. foenum-graecum* from India, seed diosgenin content in the studied genotypes ranged from 0.35% to 0.78% [88]. Paramesha et al. [36] were studied diosgenin content in the seeds of eleven varieties of *T. foenum-graecum* from India. They revealed that the plant seed is a better source of diosgenin than the plant leaf. Variations in the phytochemical traits, such as diosgenin and trigonelline content, may be attributed to the plant genotypes and environmental factors [89, 90]. In this study, we identified several species, including *T. stellata*, *T. foenum-graecum*, *T. coerulescens*, and *T. calliceras*, with high diosgenin and trigonelline content. Consequently, future research programs can be considered to explore the potential of the other wild fenugreek species to identify high-productive species and their potent populations. The populations of the studied species could serve as alternative sources for synthesizing steroid drugs in pharmaceutical industries.

## Total carotenoid, saponin, and tannin content and bitterness value

The total saponin, tannin, and carotenoid content and bitterness value are given in Table 3. The highest and lowest total carotenoid content (μg β-CARE/g DW) was obtained in TTH3 (77.29 ± 0.99) and TST2 (15.11 ± 0.11), respectively. The total carotenoid content in TSG1, TSG2, and TSG3 (73.16 ± 0.49‒76.45 ± 0.76 μg β-CARE/g DW) was also significant compared to other studied species. Total carotenoid content of the seeds of twenty-five *T. foenum-graecum* genotypes from India has been reported in the range of 1.45 to 8.54 mg/100 g [28].

Total saponin content varied broadly between species and populations (21.32 ± 0.28‒211.58 ± 1.54 mg DE/g DW). The highest total saponin content was found in TST3, TST2, TCO1, and TFG3. The total tannin content was determined in the range of 71.89 ± 0.95 to 394.63 ± 1.86 mg/100 g DW. Among the studied samples, three populations of *T. astroides* (TAS1, TAS2, and TAS3) had the highest total tannin content. The total saponin content of *T. foenum-graecum* seed from Iraq has been previously reported to be 26.65% [22]. In another study on optimizing the extraction conditions of total saponin content in *T. foenum-graecum* seed from Malaysia, the highest value was reported to be 195.89 ± 1.07 mg DE/g DW [26].

According to Abdouli et al. [31] total tannin content of *T. foenum-graecum* seed from Tunisia was determined as 2.03 and 2.59 mg/g, which is similar to our obtained results. In another study, lower content (63.69 mg/100 g) was also reported [22]. Condensed tannin and total saponin content of the seeds of twenty Omani *T. foenum-graecum* genotypes were ranged from 30.21 to 74.54 mg catechin/100 g and from 7.27 to 17.03 g/100 g, respectively [91].

TCO2 had the highest bitterness value (4.58 ± 0.12 units × 10^3^/g), while the lowest value (0.95 ± 0.01 units × 10^3^/g) was belonged to TFP1. In a study, the bitterness value (units × 10^3^/g) of two genotypes of *T. foenum-graecum* seeds from Tunisia was reported as 5.03 and 0.70 [31]. They have also been claimed that the bitterness value in vegetable foods was attributed to environmental and genetic factors. The bitterness value of *Trigonella* species and populations has not been reported so far, and this is the first report.

## Total phenol and total flavonoid content and antioxidant activity

The total phenolic and flavonoid content and antioxidant activities of the studied samples are given in Table 4. The highest TPC was obtained in TCL2 (156.23 ± 0.57 mg GAE/g DW), while the highest TFC was found in TAS2 (104.76 ± 1.67 mg/RE g DW) which was similar to the obtained results in *T. cilicica*, *T. kotschyi* Benth., and *T. cylindracea* Desv. from Turkey [38]. The highest antioxidant activity by both DPPH (15.78 ± 0.16 μg/ml) and FRAP (399.73 ± 1.32 μmol Fe^+2^/g DW) methods were obtained in TTH3 and TCL2. TPC, TFC, and antioxidant activity of fenugreeks have been widely reported [26, 35, 91–93]. Lohvina et al. [35] reported the DPPH antioxidant activity of *T. foenum-graecum* seeds from Germany as 100 μg/ml. In another study, the lowest and highest TPC in the seeds of eleven Indian *T. foenum-graecum* varieties was obtained at 91.47 to 215.55 mg/100 g FW, respectively, while, their TFC was ranged from 101.43 to 1120.83 mg/100 g FW [36]. In the current study, it was found that the seeds of the studied fenugreeks have a high TPC and TFC, which increases their antioxidant activity. According to previous studies, antioxidant activity has a direct relationship with TPC and TFC [94].

**Table 4:** Total phenol and flavonoids content, and antioxidant activity among the populations of *Trigonella* species

Populations	TPC (mg GAE/g DW)	TFC (mg RE/g DW)	DPPH (IC_50_ μg/ml)	FRAP (μmol Fe^+2^/g DW)
TAS1	107.31 ± 0.87^ab^	62.66 ± 0.96^ab^	58.37 ± 0.04^b^	227.17 ± 1.42^abc^
TAS2	129.64 ± 0.32^a^	104.76 ± 1.67^a^	31.43 ± 0.20^b^	375.66 ± 1.33^a^
TAS3	115.74 ± 0.15^ab^	75.81 ± 0.41^a^	74.67 ± 0.17^b^	211.54 ± 0.70^abcd^
TCL1	100.74 ± 1.05^ab^	55.70 ± 0.40^abc^	127.56 ± 0.26^ab^	175.33 ± 0.75^bcde^
TCL2	156.23 ± 0.57^a^	96.34 ± 0.66^a^	24.91 ± 0.08^b^	399.73 ± 1.32^a^
TCL3	52.18 ± 0.40^c^	11.63 ± 0.01^cd^	168.52 ± 0.42^a^	98.58 ± 1.12^de^
TCO1	43.92 ± 0.35^c^	15.84 ± 0.08^cd^	185.61 ± 0.24^a^	65.65 ± 0.06^e^
TCO2	92.15 ± 0.84^abc^	32.56 ± 0.03^bcd^	111.15 ± 0.09^ab^	114.97 ± 0.40^bcde^
TCO3	87.08 ± 0.09^abc^	27.63 ± 0.31^cd^	92.45 ± 0.15^ab^	143.20 ± 0.88^bcde^
TEP1	76.55 ± 0.14^bc^	32.85 ± 0.50^bcd^	152.36 ± 0.09^a^	150.79 ± 0.41^bcde^
TEP2	90.76 ± 0.77^abc^	28.33 ± 0.02^cd^	99.08 ± 0.13^ab^	165.31 ± 0.23^bcde^
TEP3	85.62 ± 0.26^abc^	25.41 ± 0.17^cd^	130.50 ± 0.16^ab^	83.14 ± 0.00^de^
TFP1	124.19 ± 1.31^a^	102.35 ± 0.40^a^	64.67 ± 0.09^b^	327.04 ± 1.06^a^
TFP2	119.72 ± 0.66^a^	76.47 ± 0.25^a^	72.21 ± 0.23^b^	256.82 ± 0.95^ab^
TFP3	95.21 ± 0.27^abc^	33.47 ± 0.05^bcd^	144.17 ± 0.16^a^	94.28 ± 0.56^de^
TFG1	49.12 ± 0.90^c^	10.72 ± 0.05^cd^	174.59 ± 0.10^a^	102.65 ± 1.02^bcde^
TFG2	67.89 ± 0.36^bc^	22.87 ± 0.08^cd^	174.72 ± 0.10^a^	72.42 ± 0.01^de^
TFG3	99.43 ± 0.15e^abc^	70.22 ± 0.77^ab^	89.66 ± 0.03^ab^	72.32 ± 0.64^de^
TSP1	69.72 ± 0.18^bc^	9.73 ± 0.15^d^	130.22 ± 0.21^ab^	78.31 ± 0.42^de^
TSP2	25.25 ± 0.04^c^	9.54 ± 0.04^d^	179.44 ± 0.08^a^	52.10 ± 0.78^e^
TSP3	72.37 ± 0.29^bc^	18.65 ± 0.17^cd^	87.16 ± 0.02^ab^	71.24 ± 0.13^de^
TST1	105.71 ± 0.08^ab^	69.19 ± 0.71^ab^	84.65 ± 0.05^ab^	252.12 ± 0.84^ab^
TST2	110.33 ± 0.94^ab^	70.54 ± 0.89^ab^	75.82 ± 0.14^ab^	325.42 ± 1.08^a^
TST3	125.76 ± 1.22^a^	95.34 ± 0.32^a^	69.47 ± 0.08^b^	245.76 ± 0.76^ab^
TSG1	92.98 ± 0.87^abc^	12.22 ± 0.03^cd^	124.44 ± 0.03^ab^	106.36 ± 0.66^bcde^
TSG2	43.24 ± 0.01^c^	7.50 ± 0.01^d^	159.24 ± 0.33^a^	70.65 ± 0.93^de^
TSG3	40.42 ± 0.02^c^	8.67 ± 0.02^d^	175.92 ± 0.19^a^	67.33 ± 0.39^e^
TTH1	122.23 ± 0.08^a^	89.36 ± 1.13^a^	77.92 ± 0.09^ab^	368.55 ± 1.29^a^
TTH2	108.21 ± 1.02^ab^	55.77 ± 0.64^abc^	77.23 ± 0.16^ab^	243.74 ± 1.26^ab^
TTH3	127.17 ± 1.45^a^	104.62 ± 1.77^a^	15.78 ± 0.16^b^	374.32 ± 1.98^a^

Each value is expressed as the mean ± SD (*n* = 3) of triplicate determinations

Means with different letters within a column are significantly different (*p* < 0.05)

For a detailed description of the plant species code, cf. Fig. 1

**Fig. 6:**
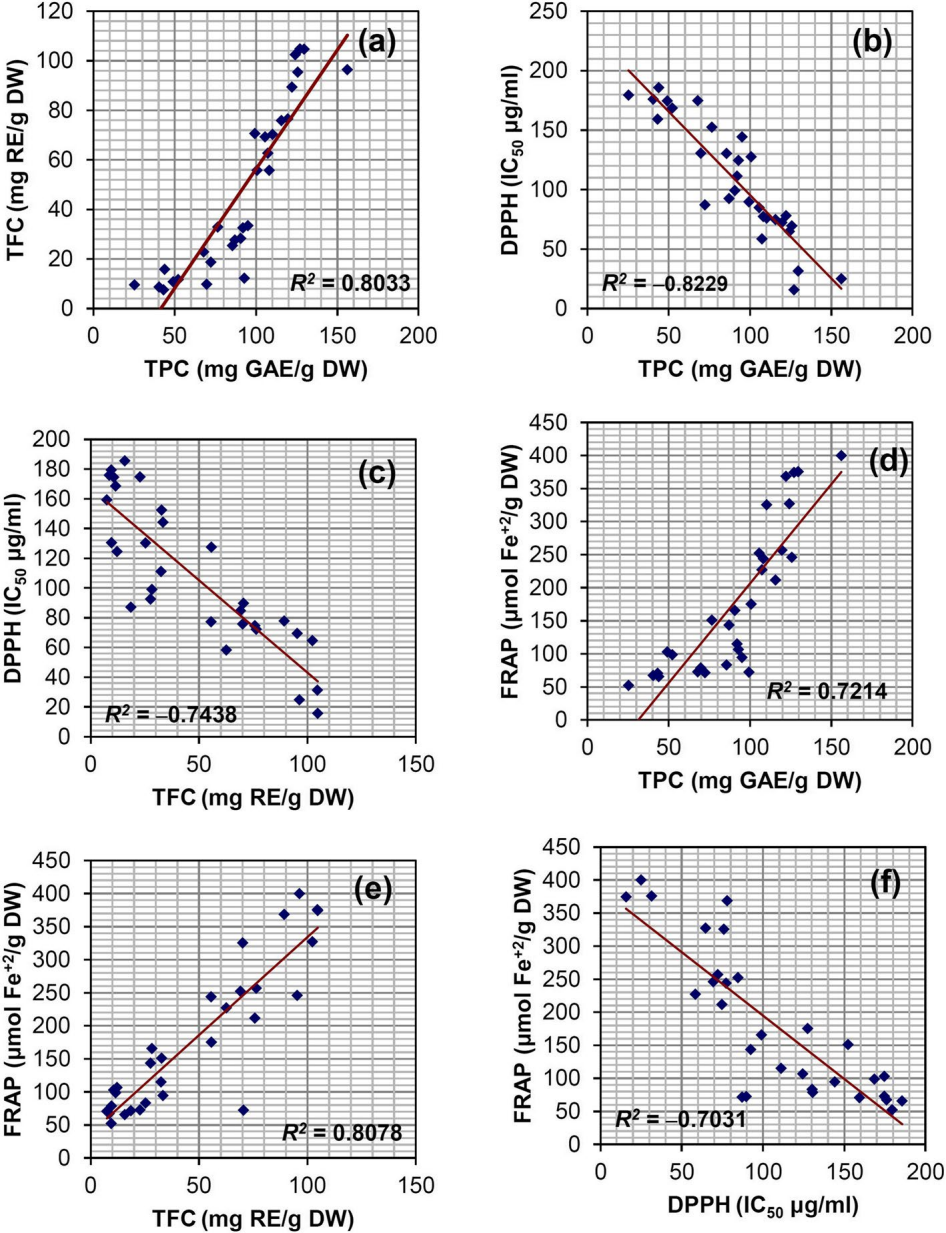
Linear correlation between total phenol and flavonoid content, and antioxidant properties (a‒f). Significant difference in 1% level

Correlation analysis showed a significant relationship between TPC and antioxidant properties by DPPH (*R2* = ‒0.8229) and FRAP (*R2* = 0.7214) in the studied fenugreeks seeds (Fig. 6a–f). In addition, TFC was positively associated with FRAP (*R2* = 0.8078) and negatively related with DPPH (*R2* = ‒0.7438), which is in agreement with earlier reports [26, 95]. The correlation of phenolic compounds is due to their reaction with all kinds of free radicals, which ultimately act as an antioxidant. The mechanism of antioxidant actions involves either hydrogen atom transfer, sequential proton loss electron transfer, and chelation of transition metals [96]. In addition, the antioxidant capacity of different extracts may not only be related to TPC and TFC, but also may be attributed to the other biochemical and phytochemical components [97]. It is also reported that genetic factors, environmental conditions, harvest time, and post-harvest processes can affect the TPC and TFC by changing the metabolic pathways and finally the biological properties including the antioxidant activity of the plant [89].

## Principal component analysis

**Fig. 7:**
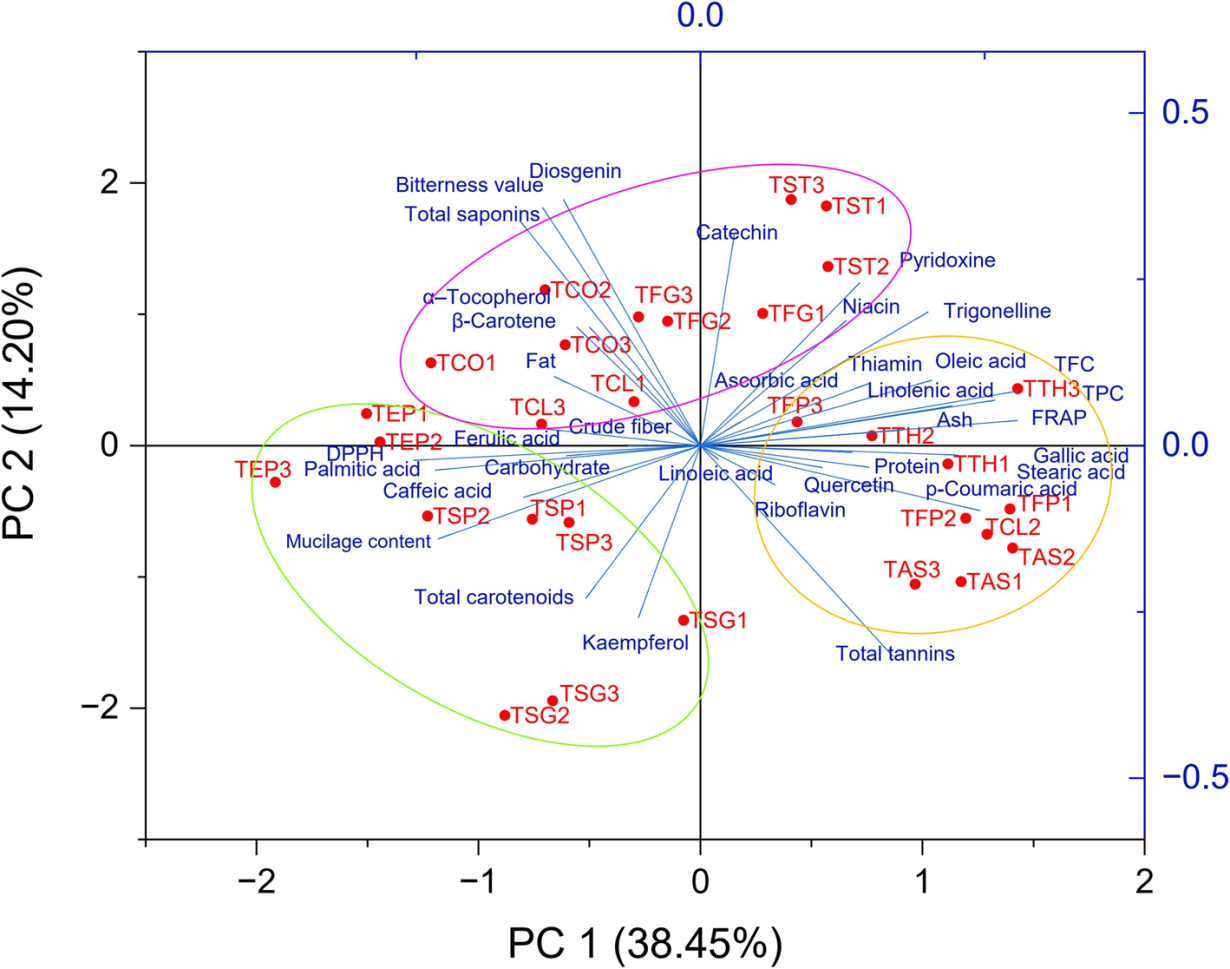
Bi-plot graph for the first and second principal components based on the major nutritional and phytochemical compounds for thirty populations of ten *Trigonella* species

Bi-plot analysis was performed using PC1 and PC2, which accounted for a total of 52.65% of the variance for major nutritional and phytochemical compounds (Fig. 7). The principal component analysis (PCA) is an efficient and dimensionality reduction method to identify similarities and dissimilarities between experimental plant genotypes based on their desired characteristics [98]. The studied *Trigonella* species and populations were divided into three groups using the measured data. The populations of *T. teheranica*, *T. filipes, T. astroides,* and TCL2 were characterized by high values in thiamine, riboflavin, *p*-coumaric acid, quercetin, gallic acid, stearic acid, oleic acid, linolenic acid, linoleic acid, ascorbic acid, trigonelline, ash, protein, total tannins, TPC, TFC, and antioxidant properties were placed in the first group, while the populations of *T. elliptica*, *T. spruneriana*, and *T. strangulata* formed the second group on the negative region and strongly contributed to kaempferol, ferulic acid, total carotenoids, palmitic acid, caffeic acid, carbohydrate, and mucilage content. The highest content of fat, crude fiber, β-carotene, α–tocopherol, catechin, pyridoxine, niacin, diosgenin, total saponins, and bitterness value was found in the populations of *T. stellata*, *T. coerulescens*, *T. foenum-graecum*, TCL1, and TCL3, which were placed them in the third group. The PCA analysis showed a significant separation with the genetics of the species and the populations with the same species are most similar to each other.

Phytochemical evaluation of plants is a suitable basis for investigating the diversity between and within plant species [99]. One of the most important reasons for the phytochemical diversity of each medicinal plant is the genetic factors, weather, and climate diversity in different regions; the existence of different climates in the world causes a significant difference in the composition of species and their different populations [100]. Many factors, including geographical conditions, differences in extraction methods, and solvents, involve in special metabolite content [101]. Reports have shown a high correlation between geographical origin and effective specialized metabolites [102].

## Conclusions

In the present study, nutritional and phytochemical traits of ten Iranian *Trigonella* species cultivated at the same environmental conditions were studied for the first time. The studied species and their populations showed a great diversity in the characteristics of macro- and micro-nutrients and plant-based bioactive compounds. *Trigonella stellata* and *T. filipes* were rich in vitamins B, E, and C, which makes them valuable for enriching diets or supplements with these essential nutrients. Some studied wild species exhibited higher protein content compared to cultivated species (*T. foenum-graecum*), suggesting their potential use in cultivation systems for consumption. Among the micro-elements, the studied seeds had a high level of iron, which can be recommended in the diet of people with iron deficiency. Some species were also rich in mucilage and can be cultivated for use in pharmaceutical industries as excipient, binder and gelling agent. The seeds of *T. stellata*, *T. filipes,* and *T. coerulescens* were suitable candidates for production oils rich in USFA. *Trigonella stellata*, *T. foenum-graecum*, and *T. coerulescens* were a good choice for providing raw materials in the production of steroid drugs.

Finally, from a practical point of view, *T. stellata*, *T. filipes, T. coerulescens*, and *T. foenum-graecum* can be selected and introduced as an adequate species for further exploitation in agricultural, food and pharmaceutical systems. Although the intrinsic factor is involved in the content of nutritional and phytochemical compounds of the studied fenugreek species, the significant variation in the compounds is also dependent on environmental factors. Therefore, the cultivation of the proposed species in other areas with different climatic conditions may cause a positive change in the content of its nutritional traits and phytochemical compounds.

### Supplementary Information

Supplementary Materials 1.

Supplementary Materials 2.

## Data Availability

All data are within the manuscript.
