# Supplementary figures and images for: Fatty acid and nutrient profiles, diosgenin and trigonelline contents, mineral composition, and antioxidant activity of the seed of some Iranian Trigonella L. species
Source: BMC Plant Biol. 2024 Jul 15;24:669. doi: 10.1186/s12870-024-05341-9 (PMC11247732; doi:10.1186/s12870-024-05341-9)

**Fig. S1** A typical chromatogram of the fatty acids from the population of TST1


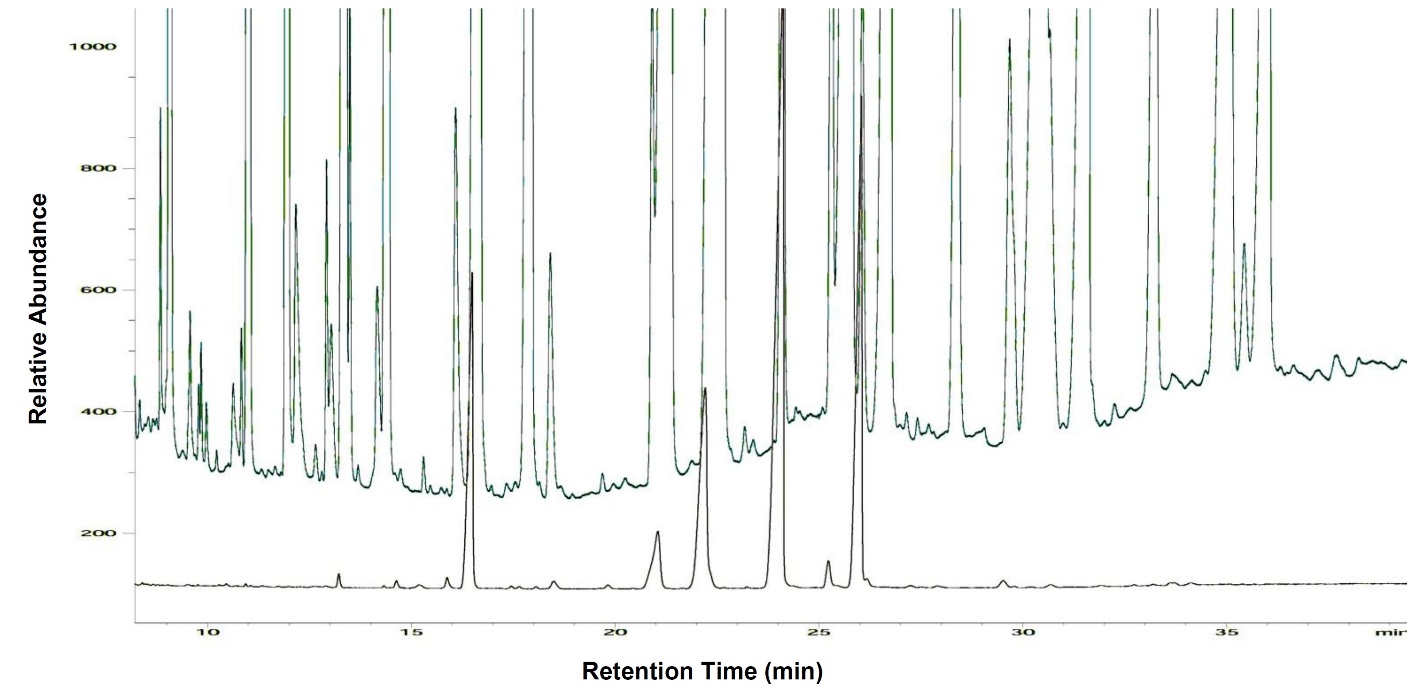

Supplement: Supplementary file 1 — Supplementary Materials 1. [file 12870_2024_5341_MOESM1_ESM.docx]
