# Supplementary material for: Fatty acid and nutrient profiles, diosgenin and trigonelline contents, mineral composition, and antioxidant activity of the seed of some Iranian Trigonella L. species
Source: BMC Plant Biol. 2024 Jul 15;24:669. doi: 10.1186/s12870-024-05341-9 (PMC11247732; doi:10.1186/s12870-024-05341-9)
Supplement: Supplementary file 2 — Supplementary Materials 2. [file 12870_2024_5341_MOESM2_ESM.docx]

**Table S1.** Information on localities investigated of thirty populations of the ten *Trigonella* species in Iran

| No. | Species | Code | Collection site | Voucher number | Geographical parameters | |
| --- | --- | --- | --- | --- | --- | --- |
|  |  |  |  |  | Latitude (N) Longitude (E) | Altitude (m) |
| 1 | *Trigonella astroides* | TAS1 | Gazvin-Soltanabad | HIAK-6566 | 36°37′, 49°65′ | 1130 |
|  |  | TAS2 | Khuzistan-Bavi | HIAK-6567 | 31°58′, 48°88′ | 25 |
|  |  | TAS3 | Ilam-Mehran | HIAK-6523 | 33°07′, 46°10′ | 136 |
| 2 | *T. calliceras* | TCL1 | Guilan-Astara | HIAK-6942 | 38°26′, 48°52′ | ‒22 |
|  |  | TCL2 | Guilan-Bandar Anzali | HIAK-6943 | 37°46′, 49°46′ | ‒26 |
|  |  | TCL3 | Mazandaran-Behshahr | HIAK-6944 | 36°71′, 53°55′ | ‒28 |
| 3 | *T. coerulescens* | TCO1 | Ardabil-Meshginshahr | HIAK-6945 | 38°40′, 47°66′ | 1400 |
|  |  | TCO2 | East Azerbaijan-Tabriz | HIAK-6946 | 38°35′, 46°30′ | 1460 |
|  |  | TCO3 | West Azerbaijan-Khoy | HIAK-6947 | 38°20′, 44°77′ | 1396 |
| 4 | *T. elliptica* | TEP1 | East Azerbaijan-Mianeh | HIAK-6529 | 37°42′, 47°72′ | 1100 |
|  |  | TEP2 | Kermanshah | HIAK-6573 | 34°23′, 47°00′ | 1374 |
|  |  | TEP3 | Kurdistan-Mariwan | HIAK-6574 | 35°52′, 46°17′ | 1320 |
| 5 | *T. filipes* | TFP1 | Ilam-Salehabad | HIAK-6531 | 33°29′, 46°11′ | 136 |
|  |  | TFP2 | Kermanshah-Qasr e Shirin | HIAK-6575 | 34°51′, 45°57′ | 333 |
|  |  | TFP3 | Lorestan-Saravand | HIAK-6530 | 49°04′, 33°29′ | 450 |
| 6 | *T. foenum-graecum* | TFG1 | Hormozgan-Minab | HIAK-6513 | 27°15′, 57°07′ | 16 |
|  |  | TFG2 | Isfahan-Ardestan | HIAK-6495 | 33°20′, 52°25′ | 1207 |
|  |  | TFG3 | Razavi Khorasan-Mashhad | HIAK-6512 | 36°20′, 59°35′ | 1065 |
| 7 | *T. spruneriana* | TSP1 | Kohgiluyeh va BoyerAhmad-Pataveh | HIAK-6583 | 30°57′, 51°16′ | 1810 |
|  |  | TSP2 | Fars-Shiraz-Dehbozorgi | HIAK-6582 | 29°63′, 52°51′ | 1500 |
|  |  | TSP3 | Zanjan-Tarom-Tashvir | HIAK-6536 | 37°13′, 48°30′ | 1638 |
| 8 | *T. stellata* | TST1 | Sistan va Baluchestan-Qasregand | HIAK-6948 | 26°32′, 60°18′ | 500 |
|  |  | TST2 | Bushehr-Borazjan | HIAK-6949 | 29°21′, 51°20′ | 80 |
|  |  | TST3 | Kerman-Kahnuj | HIAK-6950 | 29°90′, 57°66′ | 702 |
| 9 | *T. strangulata* | TSG1 | Lorestan-Khorramabad | HIAK-6951 | 33°48′, 48°35′ | 1147 |
|  |  | TSG2 | Kurdistan-Ghorveh | HIAK-6952 | 35°16′, 47°81′ | 1900 |
|  |  | TSG3 | West Azerbaijan-Urmia | HIAK-6953 | 37°55′, 45°08′ | 1363 |
| 10 | *T. teheranica* | TTH1 | Alborz-Karaj | HIAK-6954 | 36°47′, 51°42 | 2500 |
|  |  | TTH2 | Mazandaran-Chalus | HIAK-6955 | 34°40′, 51°00′ | 1120 |
|  |  | TTH3 | Tehran-Oushan | HIAK-6956 | 35°93′, 51°52′ | 1917 |
